# Surface-Alloyed Nanoporous Zinc as Reversible and Stable Anodes for High-Performance Aqueous Zinc-Ion Battery

**DOI:** 10.1007/s40820-022-00867-9

**Published:** 2022-06-14

**Authors:** Huan Meng, Qing Ran, Tian-Yi Dai, Hang Shi, Shu-Pei Zeng, Yong-Fu Zhu, Zi Wen, Wei Zhang, Xing-You Lang, Wei-Tao Zheng, Qing Jiang

**Affiliations:** 1 Key Laboratory of Automobile Materials (Jilin University), Ministry of Education, School of Materials Science and Engineering, and Electron Microscopy Center, Jilin University, Changchun, 130022 People’s Republic of China; 2 State Key Laboratory of Automotive Simulation and Control, Jilin University, Changchun, 130022 People’s Republic of China

**Keywords:** Nanoporous metal, Zinc-based alloy anode, Aqueous zinc-ion batteries, Surface alloying

## Abstract

Metallic zinc (Zn) is one of the most attractive multivalent-metal anode materials in post-lithium batteries because of its high abundance, low cost and high theoretical capacity. However, it usually suffers from large voltage polarization, low Coulombic efficiency and high propensity for dendritic failure during Zn stripping/plating, hindering the practical application in aqueous rechargeable zinc-metal batteries (AR-ZMBs). Here we demonstrate that anionic surfactant-assisted in situ surface alloying of Cu and Zn remarkably improves Zn reversibility of 3D nanoporous Zn electrodes for potential use as high-performance AR-ZMB anode materials. As a result of the zincophilic Zn_*x*_Cu_*y*_ alloy shell guiding uniform Zn deposition with a zero nucleation overpotential and facilitating Zn stripping via the Zn_*x*_Cu_*y*_/Zn galvanic couples, the self-supported nanoporous Zn_*x*_Cu_*y*_/Zn electrodes exhibit superior dendrite-free Zn stripping/plating behaviors in ambient aqueous electrolyte, with ultralow polarizations under current densities up to 50 mA cm^‒2^, exceptional stability for 1900 h and high Zn utilization. This enables AR-ZMB full cells constructed with nanoporous Zn_*x*_Cu_*y*_/Zn anode and K_*z*_MnO_2_ cathode to achieve specific energy of as high as ~ 430 Wh kg^‒1^ with ~ 99.8% Coulombic efficiency, and retain ~ 86% after long-term cycles for > 700 h.

**Supplementary Information:**

The online version contains supplementary material available at 10.1007/s40820-022-00867-9.

## Highlights

- Zn_*x*_Cu_*y*_ alloy shell was in-situ formed on self-supported three-dimensional nanoporous Zn anode by anionic surfactant-assisted surface alloying of Zn and Cu.
- The self-supported nanoporous Zn_*x*_Cu_*y*_/Zn anodes exhibit high-rate capability, outstanding reversibility and stability during Zn stripping/plating because of zincophilic Zn_*x*_Cu_*y*_ to guide uniform Zn deposition and facilitate Zn stripping.
- Aqueous Zn-ion batteries assembled with nanoporous Zn_*x*_Cu_*y*_/Zn anode and K_*z*_MnO_2_ cathode achieve specific energy of as high as ~430 Wh kg^‒1^ and retain ~86% after long-term cycles for >700 h.

**Supplementary Information:**

The online version contains supplementary material available at 10.1007/s40820-022-00867-9.

## Graphical abstract

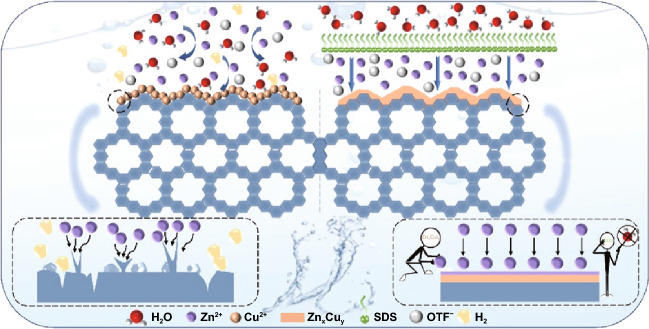

## Introduction

Highly safe and reliable cost-effective rechargeable batteries hold great promise in many emerging large-scale energy storage applications such as electric vehicles and stationary storage of intermittently available renewable energy sources (e.g., solar and wind) [1, 2]. Lithium-ion batteries as one of the most successful energy-storage devices dominate the present commercial electronics [3], but they are far from meeting future needs of grid-level energy storage due to unsustainability issues, such as high-cost and scarcity of lithium resources, and safety concerns caused by highly toxic and flammable organic electrolytes [1, 3–6]. This dilemma has raised urgent demands for developing alternative battery technologies [3–6], especially safe and low-cost aqueous rechargeable batteries based on non-lithium charge carries [7–10]. Among various attractive aqueous battery candidates [11–14], aqueous rechargeable zinc-metal batteries (AR-ZMBs) are of considerable interest because multivalent zinc metal (Zn) features high volumetric and gravimetric capacities (5854 mAh cm^‒3^ and 820 mAh g^‒1^), low Zn/Zn^2+^ redox potential (‒0.76 V versus standard hydrogen electrode, SHE), high Earth abundance and low cost [4, 9, 15–18]. Despite some high-performance cathode materials, such as manganese oxides [19–23], vanadium oxides [24–28] and quinone analogs [29, 30], have been explored to effectively accommodate Zn^2+^ via intercalation or conversion reactions, most AR-ZMBs still exhibit unsatisfactory rechargeability, hindering their practical implementation as power sources for transportation or reliable solutions for grid integration of renewable energy [4]. This is primarily caused by poor reversibility of metallic Zn anode because of its unique metallurgic characteristics and undesirable side reactions (e.g., hydrogen evolution, corrosion and by-product formation), which usually lead to large voltage polarization, dendrite formation and low coulombic efficiency (CE), during the Zn stripping/plating in ambient aqueous electrolytes [16–18, 31–33]. With an aim at improving the reversibility of Zn stripping/plating, many strategies have been proposed to tackle these irreversibility issues [34], and initial strides have been made in electrolyte modulation with additives to regulate solvation/desolvation process of Zn^2+^ [35–40] and/or electrical field distribution of Zn protuberances [41–43], crystallographic plane manipulation to guide epitaxial electrodeposition of Zn [44–46], and interface engineering of artificial solid electrolyte interphase (SEI) to inhibit side reactions [47–55]. Nevertheless, these bulk Zn metal-based anodes still undergo large voltage polarizations particularly at high current densities and thus inevitably trigger side reactions and dendrite formation during the long-term Zn stripping/plating, which result in significant compromise in rate capabilities and cycling stability of AR-ZMBs. Nanostructuring of Zn metals is one of facile strategies to depress the Zn dendrite growth and reduce the voltage polarization by making use of high specific surface area to lower local current density and improve mass transport of Zn^2+^ at electrode/electrolyte interface [56, 57]. However, nanostructured Zn metals usually are of highly chemical activity and undergo severe side reactions because of high-density low-coordination surface atoms [58, 59]. In this regard, it is highly desirable to explore novel Zn-based alloy anodes with highly compatible and stable electrode/electrolyte interfaces for high-performance AR-ZMBs.

In this work, we report self-supported three-dimensional and bicontinuous nanoporous Zn_*x*_Cu_*y*_/Zn hybrid electrodes, of which the Zn_*x*_Cu_*y*_ alloy shell is in situ formed on nanoporous Zn skeleton by anionic surfactant-assisted surface alloying of Zn and incorporated Cu, as highly reversible and dendrite-free metal anodes of AR-ZMBs. Herein, the anionic surfactant is specifically sodium dodecyl sulfate (SDS) consisting of a hydrophobic hydrocarbon tail and a hydrophilic polar headgroup. In zinc trifluoromethanesulfonate (Zn(OTF)_2_) aqueous electrolyte, these SDS molecules graft on the constituent Cu nanoparticle-decorated nanoporous Zn (Cu/Zn) and exclude free water molecules from electrode/electrolyte interface, enabling the formation of Zn_*x*_Cu_*y*_ alloy shell on nanoporous Zn skeleton during Zn stripping/plating processes. As a consequence of the zincophilic Zn_*x*_Cu_*y*_ alloy shell guiding uniform Zn deposition with a nucleation overpotential of as low as zero millivolt and the heterostructured Zn_*x*_Cu_*y*_/Zn galvanic couples to facilitate Zn stripping, the nanoporous Zn_*x*_Cu_*y*_/Zn electrodes exhibit ultralow polarizations under current densities up to 50 mA cm^‒2^ and exceptional stability for 1900 h during Zn stripping/plating in ambient aqueous electrolyte. These outstanding electrochemical properties enlist AR-ZMB full cells assembled with nanoporous Zn_*x*_Cu_*y*_/Zn anode and K_*z*_MnO_2_ cathode to achieve specific energy of ~ 430 Wh kg^‒1^ (based on the loading mass of K_*z*_MnO_2_ in the cathode) with the CE of as high as ~ 99.8% and retain ~ 86% after long-term cycling for more than 700 h.

## Experimental Section

### Materials Preparation

#### Preparation of Nanoporous Zn-Based Anodes

Precursor alloy of Zn_50_Al_50_ (at%) was firstly produced by induction melting of high-purity Zn (99.994%) and Al (99.996%) in high-purity alumina crucible and then pouring in iron casting mold with a cooling rate of ~ 10 K s^−1^ [16]. After cutting into sheets, the Zn_50_Al_50_ sheets are chemically dealloyed to prepare nanoporous Zn, in which the less-noble Al component was selectively dissolved in N_2_-purged KOH solution (1 M) [60, 61]. When rinsed in pure water and ethanol for several times, these nanoporous Zn sheets were immersed in CuCl_2_ solution (5 mM) for 15 s to obtain nanoporous Cu/Zn hybrid electrodes via a galvanic replacement reaction. The as-prepared nanoporous Cu/Zn sheets were further washed in pure water to remove residual chemical in nanopore channels and directly used as electrodes in symmetric cells. During the electrochemical Zn stripping/plating, there took place surface alloying of Zn and Cu to form Zn_*x*_Cu_*y*_/Zn core/shell structure with a three-dimensional nanoporous architecture.

#### Preparation of K_z_MnO_2_ Cathode

The K_*z*_MnO_2_ nanobelts were prepared by a modified hydrothermal method. Typically, the mixture of KMnO_4_ (40 mM) and NH_4_Cl (40 mM) in a Teflon-lined steel autoclave was heated at 150 °C for 24 h in an oil bath and magnetically stirred at a speed of 250 rpm. The K_*z*_MnO_2_-based cathode was prepared by mixing the as-prepared K_*z*_MnO_2_ nanobelts with super-P acetylene black conducting agent and poly(vinylidene difluoride) binder with a weight ratio of 70: 20: 10 in N-methyl-2-pyrrolidone (NMP), and then pasted on titanium foil with the loading mass of 1.0 mg cm^−2^.

### Physicochemical Characterizations

The microstructural and chemical features of nanoporous Zn, Cu/Zn and Zn_*x*_Cu_*y*_/Zn sheets were characterized by a field-emission scanning electron microscope equipped with an X-ray energy-dispersive spectroscopy (SEM–EDS, JEOL, JSM-7900F, 15 kV) and a field-emission transition electron microscope (TEM, JEOL, JEM-2100F, 200 kV). X-ray diffraction (XRD) measurements of all specimens were taken on a D/max2500pc diffractometer with a Cu Kα radiation. Raman spectra were measured on a micro-Raman spectrometer (Renishaw) with a 532-nm-wavelength laser at the power of 0.5 mW. X-ray photoelectron spectroscopy (XPS) analysis was conducted on a Thermo ECSALAB 250 with an Al anode. Charging effects were compensated by shifting binding energies based on the adventitious C 1* s* peak (284.8 eV). Ion concentrations in electrolytes were analyzed by inductively coupled plasma optical emission spectrometer (ICP-OES, Thermo electron).

### Electrochemical Measurements

Coin-type symmetrical cells were assembled with two identical nanoporous Cu/Zn and Zn sheets, as well as bulk Zn sheets with diameter of 1.2 cm and thickness of 100 µm, separated by a glass fiber membrane (GFM), in 1 M Zn(OTF)_2_ aqueous solution with or without 1 mM SDS. Electrochemical Zn stripping/plating behaviors of nanoporous Cu/Zn and Zn, and bulk Zn electrodes were measured at various current densities from 0.5 to 50 mA cm^−2^. Electrochemical impedance spectroscopy (EIS) measurements of as-assembled symmetric cells were taken over the frequency ranging from 100 kHz to 10 mHz with an amplitude of 10 mV at room temperature. The cycling durability tests were performed at current densities of 0.5 and 50 mA cm^−2^. The nucleation overpotentials of electrodes were investigated in a three-electrode cell in which nanoporous Cu/Zn, Zn or bulk Zn foils were employed as the working and counter electrodes, zinc wire as the reference electrode. Within 1 M Zn(OTF)_2_ with/without 1 mM SDS, chronopotentiometry measurements were taken at 0.5 mA cm^−2^. The Tafel curves were tested in the three-electrode configuration with zinc foil as the counter electrode, zinc wire as the reference electrode, and bulk Zn, nanoporous Zn, nanoporous Cu/Zn and nanoporous Zn_*x*_Cu_*y*_/Zn as the working electrodes, respectively, within Zn(OTF)_2_ aqueous electrolyte. Coin-type full zinc-ion cells were further assembled with the nanoporous Cu/Zn and Zn, bulk Zn as the anode and the titanium foil supported K_*z*_MnO_2_ as the cathode, the GFM as the separator, the 1 M Zn(OTF)_2_ solution containing with 1 mM SDS and 0.1 M Mn(OTF)_2_ as the aqueous electrolyte. The rate capability and cycling performance of full cells were carried out on a battery test system.

### Theoretical Calculation and Simulation

The density-functional theory (DFT) calculations were performed by using the Dmol3 code. The exchange–correlation potential was based on the functional of Perdew–Burke–Ernzerhof (PBE) of generalized gradient approximation (GGA). The DFT Semicore Pseudopotential (DSPP) method was employed to describe the electron–core interactions. For the basis sets, the double numerical plus polarization (DNP) was used with the real-space global orbital cutoff radius of 4.4 Å. The k-point grid was set at 4 × 4 × 1 for integrating the Brillouin zones. The structures of Zn(002), Cu(111) and Zn_*x*_Cu_*y*_(110) planes were constructed with five layers, and the bottom two layers of the atoms were fixed. The convergence criterions of the energy, maximum force, and maximum displacement were 1 × 10^–5^ Ha, 0.002 Ha Å^−1^, and 0.005 Å, respectively.

## Results and Discussion

### Characterizations of Nanoporous Hybrid Electrodes

**Fig. 1:**
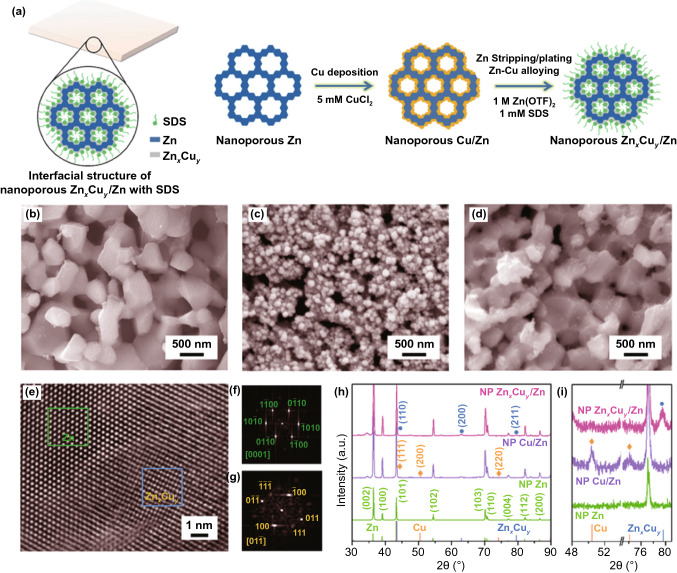
Schematic and microstructural properties of nanoporous Zn-based electrodes. **a** Schematic illustration for nanoporous shell/core Zn_*x*_Cu_*y*_/Zn sheets that are fabricated by surface alloying of Cu and Zn of Cu-decorated nanoporous Zn during sodium dodecyl sulfate (SDS)-assisted electrochemical Zn stripping/plating cycling. **b** SEM image of nanoporous Zn electrode that is prepared by chemically dealloying Zn_50_Al_50_ alloy sheets in KOH solution. **c** SEM image of nanoporous Cu/Zn hybrid electrode, in which Cu nanoparticles with diameter of ~ 50 nm are deposited on surface of nanoporous Zn skeleton via a galvanic replacement reaction. **d** SEM image of surface-alloyed nanoporous Zn_*x*_Cu_*y*_/Zn electrode after SDS-assisted Zn stripping/plating in Zn(OTF)_2_ for 10 cycles. **e** HRTEM image of Zn_*x*_Cu_*y*_/Zn interface of nanoporous Zn_*x*_Cu_*y*_/Zn electrode. **f**, **g** FFT patterns of HCP Zn (f) and cubic Zn_*x*_Cu_*y*_ (g) phases corresponding to green and blue squares in **e**. **h** Typical XRD patterns of nanoporous Zn, Cu/Zn and Zn_*x*_Cu_*y*_/Zn electrodes. The line patterns show reference cards 65-3358 and 85-1326, 02-1231 for monometallic Zn and Cu, Zn_*x*_Cu_*y*_ alloy according to JCPDS, respectively. **i** A magnification of XRD patterns of nanoporous Zn, Cu/Zn and Zn_*x*_Cu_*y*_/Zn electrodes at the characteristic diffraction peak regions of monometallic Cu and Zn_*x*_Cu_*y*_ alloy

The self-supported nanoporous Zn_*x*_Cu_*y*_/Zn electrodes are prepared by a facile procedure schematically illustrated in Fig. 1a, in which Cu nanoparticles are uniformly incorporated onto nanoporous Zn via a galvanic replacement reaction and then transformed to Zn_*x*_Cu_*y*_ alloy shell through surfactant-assisted in situ surface alloying of Cu and Zn during the Zn stripping/plating in Zn(OTF)_2_ aqueous electrolyte with SDS additive. Therein, nanoporous Zn precursor sheets are firstly fabricated by chemically dealloying Zn_50_Al_50_ alloy composed of intercross-linked hexagonal closest packed (*hcp*) Zn and face-centered cubic (*fcc*) Al phases (Fig. S1), wherein the less-noble Al one is selectively etched in a N_2_-purged KOH solution (Fig. S2) [60, 61]. Owing to the immiscibility of Al in Zn [16], selective dissolution of Al phase gives rise to interconnective pure Zn skeleton, different from traditional nanoporous metals prepared by chemically dealloying homogeneous solid-solution alloys, in which there generally remain residual less-noble elements due to parting limit effect [62, 63]. Figure 1b shows a typical SEM image of as-dealloyed nanoporous Zn, displaying a three-dimensional nanoporous architecture consisting of interpenetrative channels and interconnective Zn ligaments with characteristic length of ~ 200 nm. Because of the lowest surface energy of (002) plane [44–46], the nanoporous Zn thermodynamically prefers to expose more (002) planes. This is attested by its XRD patterns (Fig. S3a), in which the characteristic diffraction peak of (002) plane exhibits a relatively high intensity compared with the bulk Zn foil (Fig. S3a). When immersed in CuCl_2_ solution, Cu nanoparticles with diameter of ~ 50 nm are uniformly incorporated onto nanoporous Zn sheets by a galvanic replacement reaction, as displayed in the representative SEM image of as-prepared nanoporous Cu/Zn (Fig. 1c). With the assistance of SDS molecules that graft on the Cu/Zn surface and exclude free water molecules at electrode/electrolyte interface by making use of their hydrophilic polar headgroups and hydrophobic hydrocarbon tails (Fig. S4), there takes place in situ surface alloying of Cu and Zn to form Zn_*x*_Cu_*y*_ alloy shell on nanoporous Zn during the initial electrochemical cycles of Zn stripping/plating in Zn(OTF)_2_ aqueous electrolyte with SDS additive [64, 65]. Figure 1d shows a typical SEM image of nanoporous Zn_*x*_Cu_*y*_/Zn electrode after the Zn stripping/plating at 0.5 mA cm^‒2^ for 10 cycles, where the ligament surface of Zn_*x*_Cu_*y*_/Zn becomes much smoother than the rough surface of as-prepared nanoporous Cu/Zn (Fig. 1c). XPS analysis demonstrates the presence of Zn and Cu with an atomic ratio of 60/40 in the Zn_*x*_Cu_*y*_ alloy surface, in addition to the elements in the adsorbed SDS (Fig. S5). Scanning transmission electron microscopy energy-dispersive X-ray spectroscopy (STEM-EDS) elemental mapping illustrates that Cu atoms uniformly distribute along the Zn ligament (Fig. S6). Figure 1e shows a high-resolution TEM (HRTEM) image of Zn_*x*_Cu_*y*_/Zn interfacial region, revealing the seamless integration of Zn_*x*_Cu_*y*_ alloy shell on Zn core. Viewed along their < 011 > and < 0001 > zone axis, there observe two regions with distinct crystallographic structures corresponding to the cubic Zn_*x*_Cu_*y*_ and the hcp Zn, respectively, which are identified by their characteristic fast Fourier transform (FFT) patterns of the selected areas in Fig. 1e-g. XRD characterization of nanoporous Zn_*x*_Cu_*y*_/Zn electrode further verifies the hybrid structure, with two sets of diffraction patterns: the weak diffraction peaks at 2θ = 43.5°, 63.0° and 79.6° corresponding to the (110), (200) and (211) planes of cubic Zn_*x*_Cu_*y*_ in space group *Pm−3 m*(221) (JCPDS No. 02-1231), and the ones else assigned to *hcp* Zn (JCPDS No. 65-3358) (Fig. 1h), different from the initial nanoporous Cu/Zn with characteristic diffraction peaks of *fcc* Cu (JCPDS No. 85-1326) (Fig. 1g).

**Fig. 2:**
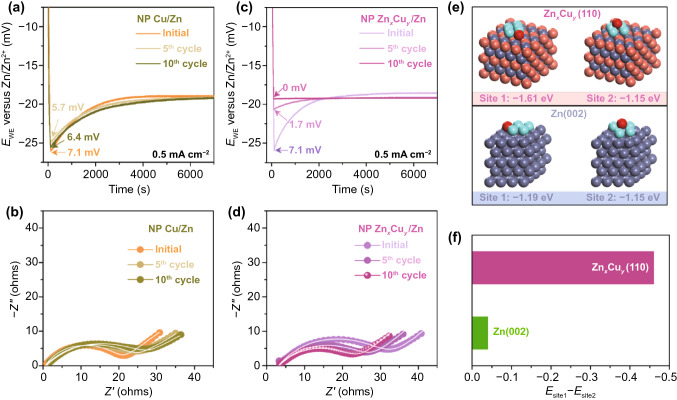
Effect of surface Zn-Cu alloying on Zn deposition. **a** Voltage–time profiles and **b** EIS spectra of galvanostatic Zn deposition on nanoporous Cu/Zn electrode at 0.5 mA cm^‒2^ after Zn stripping/plating for 0, 5 and 10 cycles without the assistance of SDS, where there does not take place alloying of Zn and Cu. **c** Voltage–time profiles and **d** EIS spectra of galvanostatic Zn deposition on nanoporous Zn_*x*_Cu_*y*_/Zn electrode after SDS-assisted Zn stripping/plating at 0.5 mA cm^‒2^ for 0, 5 and 10 cycles, which enables the formation of Zn_*x*_Cu_*y*_ alloy shell via an in situ surface alloying of Zn and Cu. The current density of galvanostatic Zn deposition: 0.5 mA cm^‒2^. **e** Zn deposition at side site (site 1) and top site (site 2) on the Zn(002) and Zn_*x*_Cu_*y*_(110) surfaces with different binding energies. **f** Comparison of energy difference between site 1 and site 2 at which Zn is deposited on the Zn(002) and Zn_*x*_Cu_*y*_(110) surfaces

The SDS-assisted in situ surface alloying of Cu and Zn is also demonstrated by XRD characterizations of nanoporous Zn_*x*_Cu_*y*_/Zn. As shown in Fig. S7a, a representative diffraction peak of cubic Zn_*x*_Cu_*y*_ appears at 2θ = 79.6° and gradually increases in intensity, along with the attenuation of Cu diffraction peaks (2θ = 50.4° and 74.1°), during the Zn stripping/plating. Owing to the SDS molecules that essentially inhibit side reactions at the electrode/electrolyte interface, metallic Zn prefers to nucleate and then deposit on Cu nanoparticles for electrochemically driven surface alloying. As demonstrated in high-resolution Zn 2*p* and Cu 2*p* XPS spectra with invariable chemical states (Fig. S8), there do not additionally produce by-products, such as general zinc oxides [9, 15–18, 31, 32]. While in the Zn(OTF)_2_ aqueous electrolyte without SDS additive, the nanoporous Cu/Zn still keeps the initial morphology (Fig. S9) and XRD patterns with characteristic diffraction peaks of individual *fcc* Cu and hcp Zn phases after the Zn stripping/plating for 10 cycles (Fig. S7b), implying that the Cu does not take part in the surface alloying with Zn. This is probably due to the by-product of zinc oxides that are generated little by little to inhibit Zn atom migration in the Zn stripping/plating processes [9, 15–18, 31, 32]. As attested by high-resolution Zn 2*p* XPS spectra (Fig. S10), the nanoporous Cu/Zn electrode has the oxidized state of surface Zn atoms to remarkably increase compared with its initial surface Zn. When the constituent Cu nanoparticles serve as the nucleation sites, the nanoporous Cu/Zn electrode exhibits the almost same galvanostatic electrodeposition behavior of metallic Zn as the initial one (Fig. 2a), with a nucleation overpotential of ~ 6.4 mV, the difference between the sharp tip voltage (−25.5 mV versus Zn/Zn^2+^) and the later stable mass-transfer-controlled overpotential (−19.1 mV) [66, 67]. Because of the lower binding energy of Cu(111) surface (Fig. S11), this value is much lower than bulk Zn foil (41.5 mV) and nanoporous Zn (17.6 mV) (Fig. S12), which are primarily composed of (101) and (002) crystal planes, respectively (Fig. S3). In sharp contrast, the nanoporous Zn_*x*_Cu_*y*_/Zn electrode has the Zn nucleation overpotential to gradually decrease to 0 mV (Fig. 2c) and substantially facilitates the nucleation and deposition of metallic Zn because of the lowest binding energy of Zn_*x*_Cu_*y*_(110) (Fig. S11). The enhanced kinetics of Zn nucleation and deposition is further demonstrated by EIS analysis of nanoporous Zn_*x*_Cu_*y*_/Zn electrode (Fig. 2d). In the Nyquist plot, their EIS spectra display single semicircles in high- and middle-frequency range and inclined lines at low frequencies during the Zn stripping/plating processes. Herein, the intersection point on the real axis at the high frequency represents the intrinsic resistance of both electrolyte and electrode (*R*_I_); the diameter of semicircle in the middle frequencies corresponds to the parallel connection of charge transfer resistance (*R*_CT_) of Zn nucleation/deposition and the constant phase element (CPE); and the slope of the inclined line at low frequencies is the Warburg resistance (*Z*_w_). Based on the equivalent circuit with these descriptors (Fig. S13a), the EIS spectra of nanoporous Zn_*x*_Cu_*y*_/Zn electrodes are analyzed using the complex nonlinear least squares fitting method. As shown in Fig. S13b, the nanoporous Zn_*x*_Cu_*y*_/Zn electrode has its *R*_CT_ to decrease to ~ 19.8 Ω from the initial value of nanoporous Cu/Zn with SDS (~ 29.7 Ω), different from nanoporous Cu/Zn without SDS additive, of which the *R*_CT_ increases due to the gradual formation of zinc oxides (Figs. 2b and S13c). Furthermore, the Zn atoms thermodynamically prefer to deposit evenly and parallel to the Zn_*x*_Cu_*y*_ alloy surface, effectively inhibiting the formation of Zn dendrites. As illustrated by DFT calculations (Fig. 2e), the Zn_*x*_Cu_*y*_(110) surface could afford a special deposition location at the side site (site 1) of early stage with an adsorption energy of as low as ~ −1.61 eV, ~ 0.46 eV lower than the adsorption energy (~ −1.15 eV) at the top site (site 2) (Fig. 2f). This implies a more favorable horizontal growth of Zn on Zn_*x*_Cu_*y*_ alloy surface relative to the monometallic Zn(002) plane that features a smooth equipotential surface and compact structure with the energy difference of ~ 0.04 eV for Zn deposition locations at site 1 and site 2 (Fig. 2e) [44–46]. Owing to the Zn_*x*_Cu_*y*_ alloy shell that can alleviate the Zn corrosion, the nanoporous Zn_*x*_Cu_*y*_/Zn electrode exhibits a more positive corrosion potential (− 10 mV) and a smaller corrosion current density (3 μA cm^−2^) than nanoporous Cu/Zn (− 18 mV, 7 μA cm^−2^), nanoporous Zn (− 36 mV, 9 μA cm^−2^) electrodes (Fig. S14).

### Electrochemical Properties of Nanoporous Zn-based Electrodes

**Fig. 3:**
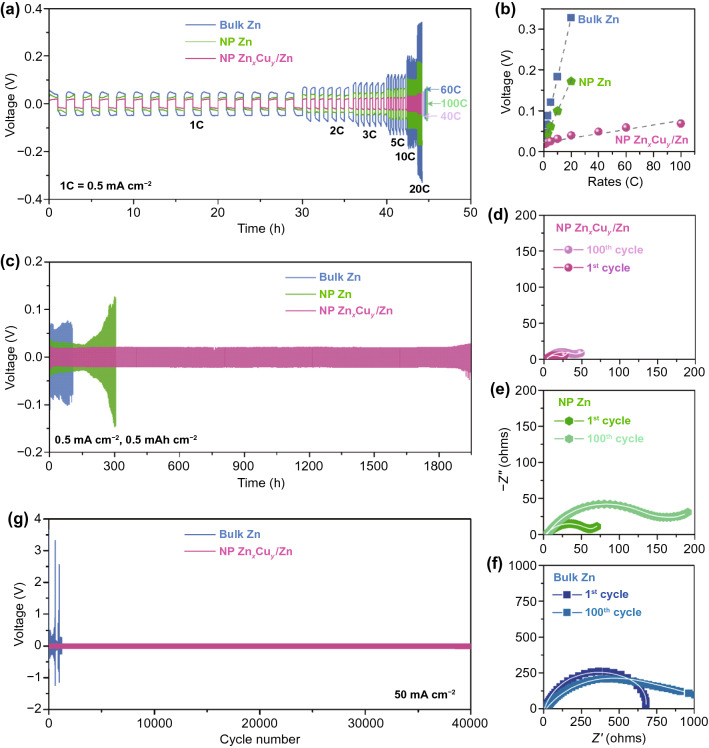
Electrochemical performance of symmetric cells. **a** Voltage profiles of nanoporous Zn_*x*_Cu_*y*_/Zn symmetric cell at various rates from 1 to 100C (1C = 0.5 mA cm^‒2^) in 1 M Zn(OTF)_2_ with 1 mM SDS, comparing with those of symmetric batteries of bulk Zn and nanoporous Zn electrodes in 1 M Zn(OTF)_2_. **b** Overpotentials of Zn stripping/plating for symmetric cells based on nanoporous Zn_*x*_Cu_*y*_/Zn and nanoporous Zn, bulk Zn in 1 M Zn(OTF)_2_ with/without 1 mM SDS as a function of rate. **c** Long-term cycling stability of Zn stripping/plating for symmetric cells based on nanoporous Zn_*x*_Cu_*y*_/Zn and nanoporous Zn, bulk Zn in 1 M Zn(OTF)_2_ with/without 1 mM SDS.EIS spectra of **d** nanoporous Zn_*x*_Cu_*y*_/Zn, **e** nanoporous Zn and **f** bulk Zn symmetric cells before and after 100 cycles of stripping/plating in 1 M Zn(OTF)_2_ with/without 1 mM SDS. **g** Long-term Zn stripping/plating stability of symmetric cells based on nanoporous Zn_*x*_Cu_*y*_/Zn and bulk Zn electrodes in 1 M Zn(OTF)_2_ with/without 1 mM SDS at 50 mA cm^‒2^, respectively

To investigate the Zn stripping/plating behaviors of nanoporous Zn_*x*_Cu_*y*_/Zn electrode, electrochemical measurements are carried out on its symmetric cell in 1 M Zn(OTF)_2_ aqueous electrolyte with SDS additive. Figure 3a shows the voltage profiles of symmetric nanoporous Zn_*x*_Cu_*y*_/Zn cell during the Zn stripping/plating processes at various rates from 1 to 100C, comparing with those of symmetric ones based on monometallic nanoporous Zn and bulk Zn electrodes in 1 M Zn(OTF)_2_ aqueous electrolyte without SDS additive, respectively. Here 1C represents a one-hour stripping and plating at the current density of 0.5 mA cm^‒2^. Evidently, the symmetric nanoporous Zn_*x*_Cu_*y*_/Zn cell exhibits relatively flat and symmetric voltage plateaus with an absolute overpotential of ~ 19 mV at 1C rate, ~ 17 and ~ 28 mV lower than those of the symmetric nanoporous Zn (~ 36 mV) and bulk Zn (~ 47 mV) ones. This observation implies the synergistic effects of chemical and structural features in nanoporous Zn_*x*_Cu_*y*_/Zn electrode, i.e., the highly zincophilic Zn_*x*_Cu_*y*_ alloy shell and the large electroactive surface area [68, 69], which facilitate the Zn stripping and plating with a low voltage polarization. As the rate increases to 5C, 10C and 20C, the nanoporous Zn_*x*_Cu_*y*_/Zn cell displays steadily increasing voltage hysteresis of ~ 7, ~ 13 and ~ 21 mV, much lower than the symmetric ones based on nanoporous Zn (~ 26, ~ 73 and ~ 146 mV) and bulk Zn (~ 73, ~ 135 and ~ 280 mV). Even the current density further increases to 50 mA cm^‒2^ (100C), the nanoporous Zn_*x*_Cu_*y*_/Zn cell still has an overpotential of as low as ~ 69 mV, comparable to the nanoporous Zn cell at 2.5 mA cm^‒2^ (~ 62 mV) (Fig. 3b). In view that both nanoporous Zn_*x*_Cu_*y*_/Zn and Zn electrodes have almost the same nanoporous architecture, the superior rate capability of nanoporous Zn_*x*_Cu_*y*_/Zn electrode highlights the significant role of Zn_*x*_Cu_*y*_ alloy shell in substantially boosting kinetics of Zn nucleation and deposition. This expectation is further attested by their distinct EIS spectra (Fig. S15a), where the symmetric nanoporous Zn_*x*_Cu_*y*_/Zn cell has the lowest *R*_CT_ value (Fig. S15b).

Figure 3c compares the long-term Zn stripping/plating cycling stabilities of nanoporous Zn_*x*_Cu_*y*_/Zn and monometallic nanoporous Zn and bulk Zn electrodes in their symmetric cells, which are performed at the rate of 1C with a constant areal capacity of 0.5 mAh cm^‒2^. As a consequence of lowering the current density by increasing the electroactive surface area, nanoporous Zn effectively alleviates the voltage polarization of Zn stripping/plating [68, 69]. This enlists the symmetric nanoporous Zn cell to retain stable voltage profile until for 155 h, outperforming symmetric bulk Zn cell that as usual displays violent fluctuation and high overpotential in a short lifetime. When extending the cycling time, there take place severe side reactions of Zn oxidation on nanoporous Zn electrode, which dramatically increases the voltage polarization and then leads to an abrupt failure. Owing to the presence of Zn_*x*_Cu_*y*_ alloy shell that effectively guides the reversible and dendrite-free Zn stripping/plating, the symmetric nanoporous Zn_*x*_Cu_*y*_/Zn cell maintains extremely stable voltage profiles at 0.5 mA cm^‒2^ for more than 1,900 h, outperforming the monometallic nanoporous Zn. The excellent stability of nanoporous Zn_*x*_Cu_*y*_/Zn is further confirmed by the negligible change of EIS spectra before and after the Zn stripping/plating for 100 cycles (Fig. 3d). Therein, the *R*_I_ and *R*_CT_ values only increase by ~ 1 Ω and ~ 12 Ω, much smaller than those of monometallic nanoporous Zn (~ 2 Ω, ~ 91 Ω) and bulk Zn (~ 12 Ω, ~ 236 Ω) with severe side reactions of Zn oxidations (Fig. S16). As revealed by Raman spectrum of nanoporous Zn_*x*_Cu_*y*_/Zn after the long-term cycling measurement of Zn stripping/plating (Fig. S17a), there do not display additional Raman bands, in addition to the characteristic ones of ZnO at ~ 381, ~ 437, ~ 580 and ~ 1100 cm^‒1^ with the almost constant intensities [70]. These observations are different from the observations in nanoporous Zn and bulk Zn electrodes (Fig. S17b-c), where there appear neoformative Raman bands at 252, 305 and 837 cm^‒1^ corresponding to Zn_*x*_(OTF)_*y*_(OH)_2*x−y*_·*n*H_2_O [71], in addition to more intensive Raman bands of ZnO at ~ 381, ~ 437, ~ 580 and ~ 1100 cm^‒1^. The superior stability of nanoporous Zn_*x*_Cu_*y*_/Zn electrode is further illustrated by the almost same nanoporous structure after 500 cycles (1000 h) as the initial one (Fig. S18a). This observation is in sharp contrast with monometallic nanoporous Zn (Fig. S18b) and bulk Zn electrodes (Fig. S18c), which undergoes severe dendrites growth and cracks when only performing for 150 cycles (300 h) and 50 cycles (100 h), respectively. Even at the rate of as high as 100C (50 mA cm^‒2^) (Fig. 3g), the nanoporous Zn_*x*_Cu_*y*_/Zn does not display evident voltage fluctuation for more than 40,000 cycles of Zn stripping/plating along with the energy efficiency of ~ 99.9% (Fig. S19).

### Electrochemical Performance of AR-ZMB Full Cells

**Fig. 4:**
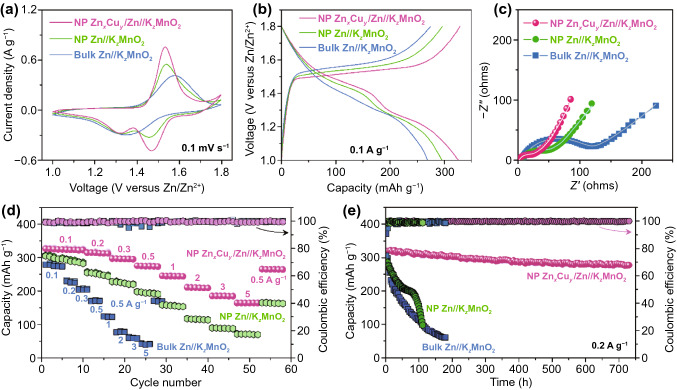
Electrochemical performance of full Zn-ion cells. **a** Representative CV curves of full cells of nanoporous Zn_*x*_Cu_*y*_/Zn//K_z_MnO_2_, nanoporous Zn//K_*z*_MnO_2_ and bulk Zn//K_*z*_MnO_2_. Scan rate: 0.1 mV s^‒1^. **b** Representative charge/discharge voltage profiles of nanoporous Zn_*x*_Cu_*y*_/Zn//K_*z*_MnO_2_, nanoporous Zn//K_*z*_MnO_2_ and bulk Zn//K_*z*_MnO_2_ full cells at the specific current of 0.1 A g^‒1^ (based on the loading mass of electroactive K_*z*_MnO_2_ at the cathode). **c** EIS spectra of nanoporous Zn_*x*_Cu_*y*_/Zn//K_*z*_MnO_2_, nanoporous Zn//K_*z*_MnO_2_ and bulk Zn//K_*z*_MnO_2_ full cells. **d** Comparisons of rate performance and coulombic efficiency for nanoporous Zn_*x*_Cu_*y*_/Zn//K_*z*_MnO_2_, nanoporous Zn//K_*z*_MnO_2_ and bulk Zn//K_*z*_MnO_2_ full cells, where are performed at various specific currents from 0.1 to 5 A g^‒1^. **e** Capacity retentions and coulombic efficiencies of nanoporous Zn_*x*_Cu_*y*_/Zn//K_*z*_MnO_2_, nanoporous Zn//K_*z*_MnO_2_ and bulk Zn//K_*z*_MnO_2_ full cells in a long-term charge/discharge cycling measurements at the specific current of 0.2 A g^‒1^

Full AR-ZMB cells are assembled with nanoporous Zn_*x*_Cu_*y*_/Zn electrode as the anode and K^+^-preintercalated α-MnO_2_ (K_*z*_MnO_2_) nanobelts as the cathode, a mixture solution of 1 M Zn(OTF)_2_, 0.1 M Mn(OTF)_2_ and 1 mM SDS as the aqueous electrolyte (nanoporous Zn_*x*_Cu_*y*_/Zn//K_*z*_MnO_2_). Therein, the K_*z*_MnO_2_ nanobelts are prepared by a modified hydrothermal method (Fig. S20) and then mixed with super-P acetylene black conducting agent and poly(vinylidene difluoride) binder on titanium foil [16, 72]. Figure 4a shows a representative cyclic voltammetry (CV) curve of full AR-ZMB device of nanoporous Zn_*x*_Cu_*y*_/Zn//K_*z*_MnO_2_ at a scan rate of 0.1 mV s^‒1^, displaying primary redox peaks at 1.55 and 1.47 V that correspond to the intercalation/deintercalation of Zn^2+^ [16, 19–22]. Although the nanoporous Zn_*x*_Cu_*y*_/Zn//K_*z*_MnO_2_ AR-ZMB has the same K_*z*_MnO_2_-based cathode material as the nanoporous Zn//K_*z*_MnO_2_ and bulk Zn//K_*z*_MnO_2_ ones, it exhibits superior voltammetric behaviors, with a higher current density and a smaller redox peak voltage difference, at various scan rates from 0.1 to 5 mV s^‒1^ (Fig. S21). These observations imply the significant role of nanoporous Zn_*x*_Cu_*y*_/Zn hybrid anode in improving rate capability of AR-ZMBs by virtue of the synergic effect of Zn_*x*_Cu_*y*_ alloy shell and nanoporous architecture on Zn stripping/plating kinetics. Figure S22a-c shows the voltage profiles for the galvanostatic charge and discharge of nanoporous Zn_*x*_Cu_*y*_/Zn//K_*z*_MnO_2_, nanoporous Zn//K_*z*_MnO_2_ and bulk Zn//K_*z*_MnO_2_ AR-ZMBs at various specific currents from 0.1 to 5 A g^‒1^, displaying obvious voltage plateaus that are consistent with the corresponding redox peaks in their corresponding CV curves (Fig. S21a-c). Here the applied specific current and the achieved capacity are calculated by the loading mass of K_*z*_MnO_2_ in the cathode. Evidently, the use of nanoporous Zn_*x*_Cu_*y*_/Zn anode not only increases the charge/discharge capacities but also improves the energy efficiency of AR-ZMB by lowering the voltage polarization. As shown in Fig. 4b, the discharge capacity of nanoporous Zn_*x*_Cu_*y*_/Zn//K_*z*_MnO_2_ AR-ZMB can reach ~ 325 mAh g^‒1^ at the specific current of 0.1 A g^‒1^, higher than those of nanoporous Zn//K_*z*_MnO_2_ (~ 295 mAh g^‒1^) and bulk Zn//K_*z*_MnO_2_ ones (~ 268 mAh g^‒1^). Even as the specific current increases to 5 A g^‒1^, the nanoporous Zn_*x*_Cu_*y*_/Zn//K_*z*_MnO_2_ AR-ZMB still achieves the charge/discharge capacities of ~ 165/ ~ 164 mAh g^‒1^, with a Coulombic efficiency of as high as ~ 99.2% (Fig. 4d), ~ 2.3- and ~ 3.8-fold higher than the values of nanoporous Zn//K_*z*_MnO_2_ (~ 71/ ~ 71 mAh g^‒1^) and bulk Zn//K_*z*_MnO_2_ ones (~ 42/ ~ 40 mAh g^‒1^). The superior rate capability of nanoporous Zn_*x*_Cu_*y*_/Zn//K_*z*_MnO_2_ AR-ZMB is also demonstrated by the EIS spectrum in the Nyquist plot (Fig. 4c), where the *R*_CT_ value is only ~ 14 Ω, much lower than those of nanoporous Zn//K_*z*_MnO_2_ (~ 38 Ω) and bulk Zn//K_*z*_MnO_2_ (~ 103 Ω) ones, respectively (Fig. S23). When increasing Zn utilization in nanoporous Zn_*x*_Cu_*y*_/Zn electrode to ~ 37.6%, the overall energy density of nanoporous Zn_*x*_Cu_*y*_/Zn//K_*z*_MnO_2_ full cell can reach ~ 204 Wh kg^−1^ (Fig. S24). The self-discharge performance of nanoporous Zn_*x*_Cu_*y*_/Zn//K_*z*_MnO_2_ cell is shown in Fig. S25. The voltage of the cell evidently drops to 1.531 V in ~ 15 h, which is due to the pseudocapacitive discharge behavior. While in the subsequent ~ 485 h, the nanoporous Zn_*x*_Cu_*y*_/Zn//K_*z*_MnO_2_ cell exhibits a very low self-discharge rate (0.18 mV h^−1^) because of ultralow insertion kinetics of Zn^2+^ [16]. Owing to the highly zincophilic Zn_*x*_Cu_*y*_ alloy shell guiding the reversible and dendrite-free Zn stripping/plating, the nanoporous Zn_*x*_Cu_*y*_/Zn//K_*z*_MnO_2_ AR-ZMB also exhibits exceptional long-term stability during the galvanostatic charge/discharge cycling measurements. As shown in Fig. 4e, it achieves initial charge/discharge capacities of ~ 320/~ 319 mAh g^‒1^ (i.e., specific energy of ~ 430 Wh kg^‒1^) at 0.2 A g^‒1^ and still retains ~ 86% (~ 278/~ 278 mAh g^‒1^) after more than 700 h, along with the Coulombic efficiency of as high as ~ 99.8%. Even at the specific current of as high as 1 A g^‒1^, the capacity retention of nanoporous Zn_*x*_Cu_*y*_/Zn//K_*z*_MnO_2_ AR-ZMB can reach ~ 84% after 800 cycles. However, the nanoporous Zn//K_*z*_MnO_2_ and bulk Zn//K_*z*_MnO_2_ devices undergo fast capacity degradation in 100 h probably due to the poor reversibility of monometallic Zn (Figs. 4e and S26).

## Conclusions

In summary, we have developed three-dimensional and bicontinuous nanoporous Zn_*x*_Cu_*y*_/Zn hybrid electrodes for the use as highly reversible and dendrite-free Zn anode in aqueous rechargeable zinc-metal batteries. By making use of amphiphilic properties of SDS, there form a hydrophilic SDS/Cu/Zn interface to substantially inhibit side reactions and thus facilitate in situ surface alloying of Cu and Zn during Zn stripping/plating. Owing to the nanoporous architecture to reduce the current density per electrochemical surface area and the Zn_*x*_Cu_*y*_ alloy shell to guide uniform and horizontal Zn deposition with a zero millivolt nucleation overpotential and facilitate Zn stripping via the formation of Zn_*x*_Cu_*y*_/Zn galvanic couples, the symmetric nanoporous Zn_*x*_Cu_*y*_/Zn cell exhibits highly reversible and dendrite-free Zn stripping/plating behaviors in 1 M Zn(OTF)_2_ aqueous electrolyte, with ultralow polarizations and stable voltage profile under various current densities up to 50 mA cm^‒2^. It maintains stable Zn stripping/plating for as long as 1900 h at 0.5 mA cm^‒2^ and for 40,000 cycles at 50 mA cm^‒2^, respectively, outperforming the symmetric cells based on monometallic nanoporous Zn and bulk Zn. These outstanding electrochemical properties enlist AR-ZMB full cells with nanoporous Zn_*x*_Cu_*y*_/Zn anode and K_*z*_MnO_2_ cathode to achieve specific energy of ~ 430 Wh kg^‒1^ (based on the loading mass of K_*z*_MnO_2_ in the cathode) with the Coulombic efficiency of as high as ~ 99.8% and the retention of ~ 86% for more than 700 h.

## Supplementary Information

Below is the link to the electronic supplementary material.

Supplementary file1 (PDF 2923 kb)
