# Supplementary material for: Surface-Alloyed Nanoporous Zinc as Reversible and Stable Anodes for High-Performance Aqueous Zinc-Ion Battery
Source: Nanomicro Lett. 2022 Jun 14;14:128. doi: 10.1007/s40820-022-00867-9 (PMC9198195; doi:10.1007/s40820-022-00867-9)
Supplement: Supplementary file 1 — Supplementary file1 (PDF 2923 kb) [file 40820_2022_867_MOESM1_ESM.pdf]

Supporting Information for

Surface-Alloyed Nanoporous Zinc as Reversible and Stable Anodes for High-Performance Aqueous Zinc-Ion Battery

Huan Meng[1, #], Qing Ran[1, #], Tian-Yi Dai[1, #], Hang Shi[1], Shu-Pei Zeng[1], Yong-Fu Zhu[1], Zi Wen[1], Wei Zhang[1], Xing-You Lang[1, 2, *], Wei-Tao Zheng[1], Qing Jiang[1, *]

[1]Key Laboratory of Automobile Materials (Jilin University), Ministry of Education, School of Materials Science and Engineering, and Electron Microscopy Center, Jilin University, Changchun 130022, P. R. China

[2]State Key Laboratory of Automotive Simulation and Control, Jilin University, Changchun 130022, P. R. China

[#] Huan Meng, Qing Ran and Tian-Yi Dai have contributed equally to this work

*Corresponding authors. E-mail: xylang@jlu.edu.cn (Xing-You Lang), jiangq@jlu.edu.cn (Qing Jiang)

Supplementary Figures

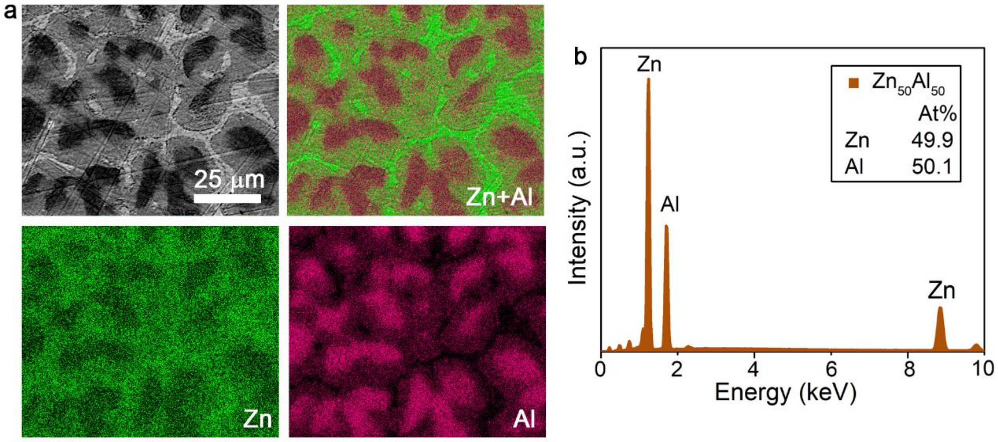

Fig. S1 (**a**) Low-magnification SEM backscattered electron image of $Zn_{50}Al_{50}$ precursor alloy and the corresponding EDS elemental mapping of Zn and Al, which are in green and purple, respectively. (**b**) EDS spectrum of $Zn_{50}Al_{50}$ precursor alloy

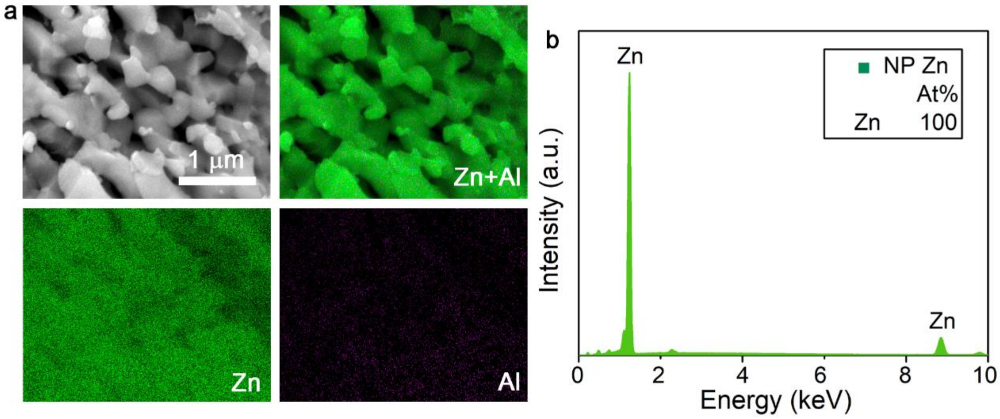

Fig. S2 (**a**) Low-magnification SEM backscattered electron image of as-prepared nanoporous Zn sheets and the corresponding EDS elemental mapping of Zn and Al, which are in green and purple, respectively. (**b**) EDS spectrum of as-prepared nanoporous Zn sheets

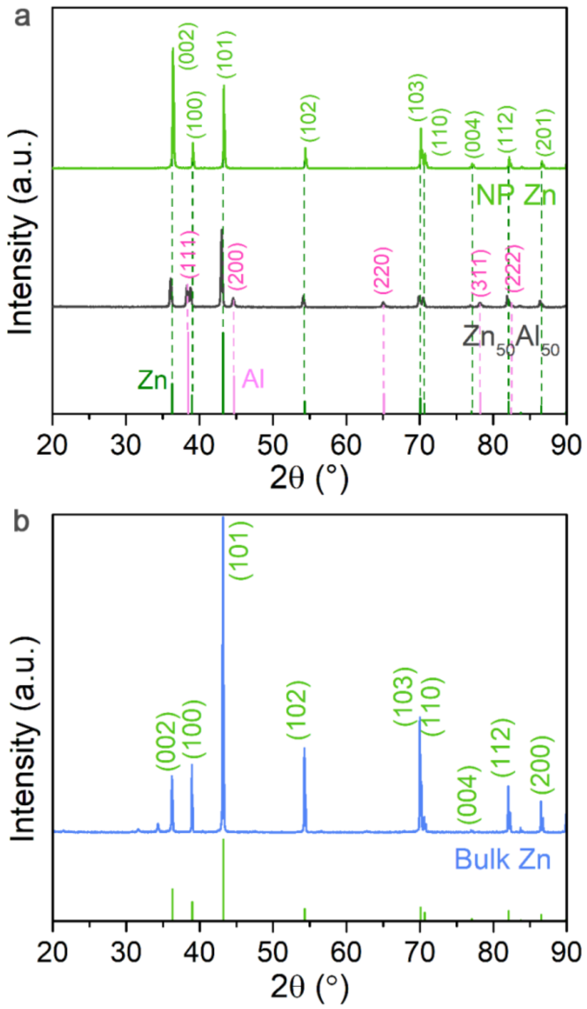

Fig. S3 (**a**) XRD patterns of $Zn_{50}Al_{50}$ precursor alloy and as-dealloyed nanoporous Zn in N_2-purged 1 M KOH solution. Therein, the nanoporous Zn is primarily composed of (002) plane. (**b**) XRD patterns of bulk Zn foil, which is primarily composed of (101) crystal plane

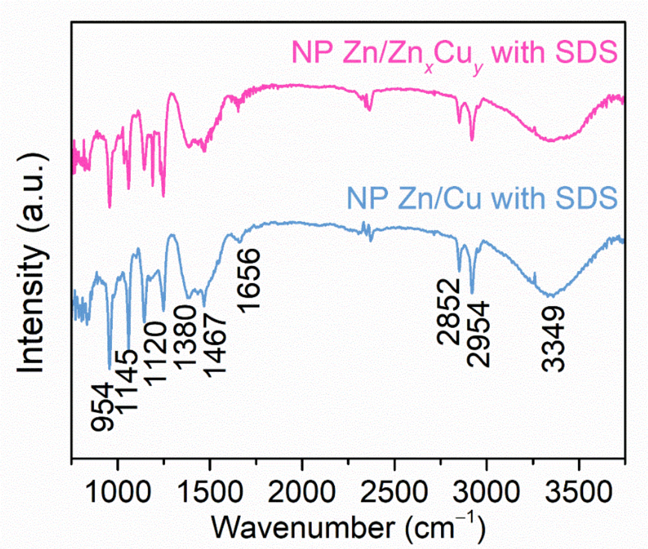

Fig. S4 FTIR spectra of SDS grafted on nanoporous Zn/Cu and Zn_xCu_y/Zn. The peak at 954 cm^{-1} corresponds to the asymmetric stretching vibration of C-O-S of SDS. The peaks at 1380 and 1467 cm^{-1} are symmetric and asymmetric deformation vibrations of $-CH_3$, respectively. The peaks at 2852 and 2954 cm^{-1} are due to $-CH_2$ symmetric and asymmetric stretching

vibrations respectively. In addition, the peaks at 1656 and 3349 cm^{-1} are assigned to the characteristic peaks of water molecules adsorbed on the surface. Compared with those of SDS grafted on nanoporous Zn/Cu, the peaks of the SDS on nanoporous Zn$_x$Cu$_y$/Zn in the range of 700-1500 cm^{-1} blue shift, indicating the charge transfer between the hydrophilic polar headgroup of SDS and the Zn$_x$Cu$_y$ alloy. Meanwhile, in the FTIR spectrum of nanoporous Zn$_x$Cu$_y$/Zn with SDS, new peaks corresponding to S=O stretching vibration and CF$_3$ asymmetric stretching vibration in OTF$^-$ appear at 1033 and 1189 cm^{-1}, respectively, due to the adsorption of OTF$^-$ on the electrode surface during the cycling process. The peak of the two curves at 1145 cm^{-1} may be attributed to the deviation of SO_4^{2-} characteristic peak of SDS head at 1220 cm^{-1}

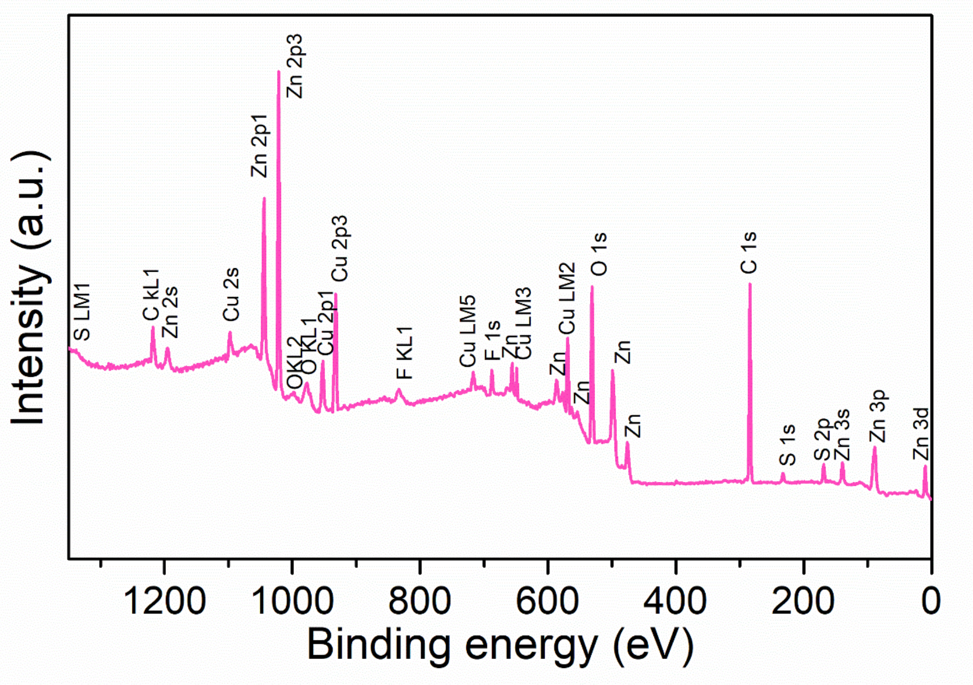

Fig. S5 XPS survey spectrum of nanoporous Zn$_x$Cu$_y$/Zn electrode

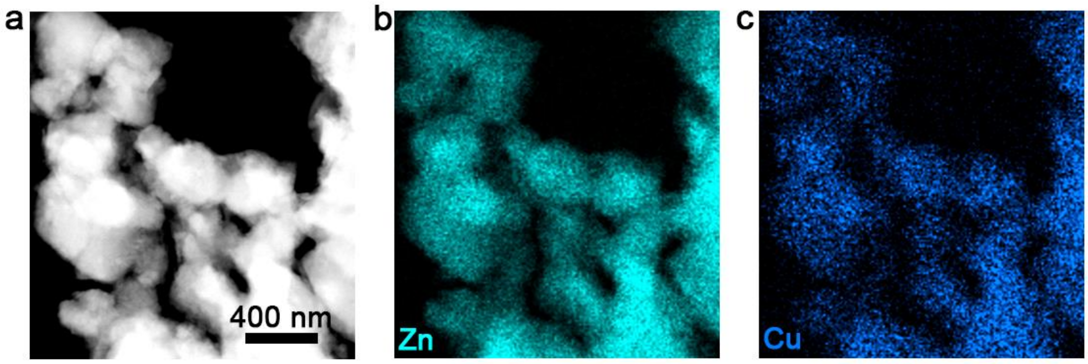

Fig. S6 (**a**) STEM image and the corresponding (**b**) Zn and (**c**) Cu EDS elemental mapping of nanoporous Zn$_x$Cu$_y$/Zn electrode, with Zn in cyan and Cu in blue

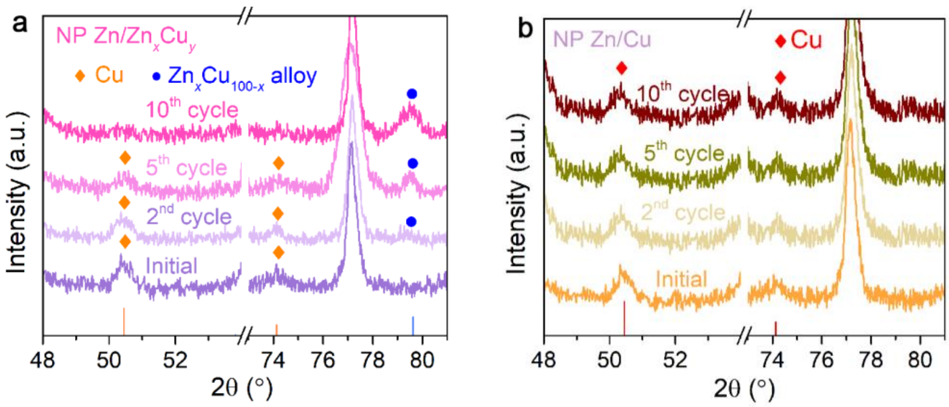

Fig. S7 (**a**) Typical XRD patterns of nanoporous Zn_xCu_y/Zn, of which the Zn_xCu_y alloy shell in-situ forms on Zn core via surface alloying of Cu and Zn with the assistance of SDS during Zn stripping/plating processes. (**b**) Typical XRD patterns of nanoporous Cu/Zn, which keeps the initial Cu/Zn structure during Zn stripping/plating processes without the assistance of SDS

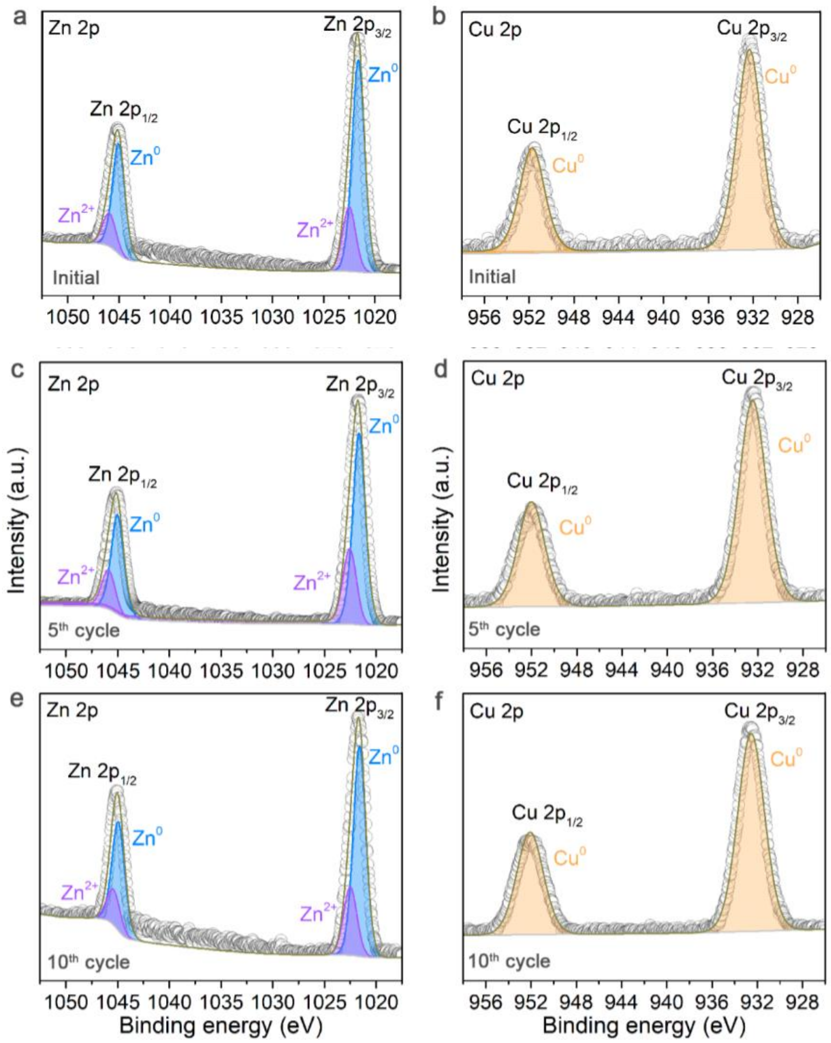

Fig. S8 High-resolution XPS spectra of Zn 2p of nanoporous Zn_xCu_y/Zn during the Zn stripping/plating for 0 (**a**), 5 (**c**) and 10 (**e**) cycles **e** in 1 M $Zn(OTF)_2$ aqueous electrolyte with SDS additive. High-resolution XPS spectra of Cu 2p of nanoporous Zn_xCu_y/Zn during the Zn stripping/plating for 0 (**b**), 5 (**d**) and 10 (**f**) cycles in 1 M $Zn(OTF)_2$ aqueous electrolyte with SDS additive

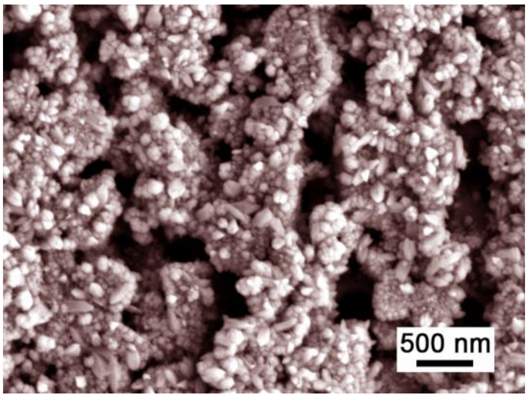

Fig. S9 Representative SEM image of nanoporous Cu/Zn after Zn stripping/plating for 10 cycles in 1 M Zn(OTF)$_2$ without SDS additive

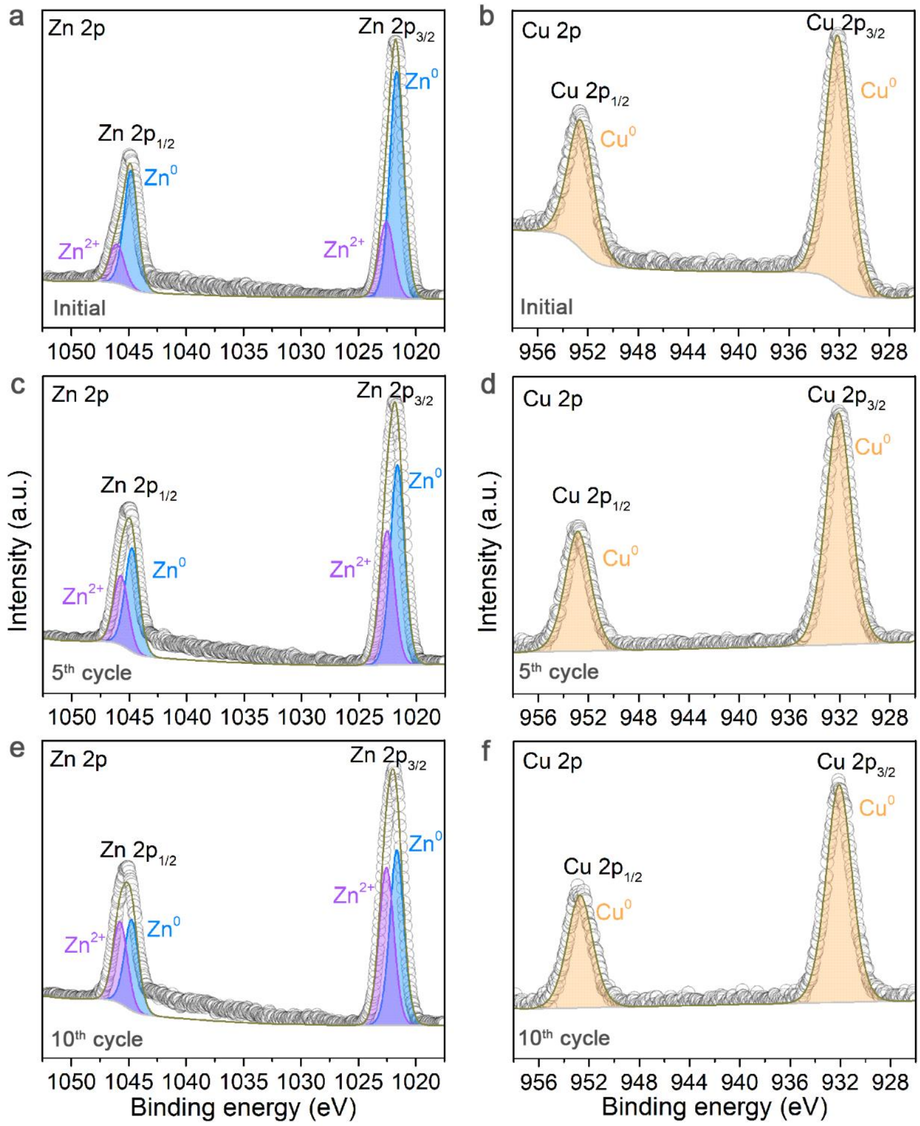

Fig. S10 (**a**, **c**, **e**) High-resolution XPS spectra of Zn 2p of nanoporous Cu/Zn during the Zn stripping/plating for 0 (**a**), 5 (**c**) and 10 cycles (**e**) in 1 M Zn(OTF)$_2$ aqueous electrolyte without SDS additive. (**b**, **d**, **f**) High-resolution XPS spectra of Cu 2p of nanoporous Cu/Zn during the Zn stripping/plating for 0 (**b**), 5 (**d**) and 10 (**f**) cycles in 1 M Zn(OTF)$_2$ aqueous electrolyte without SDS additive

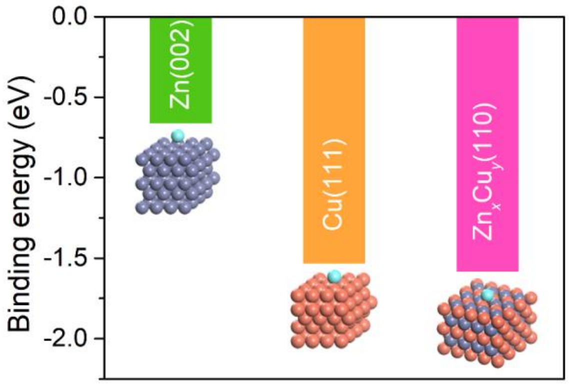

Fig. S11 Comparison of binding energy for Zn atom deposition on Zn(002), Cu(111) and Zn_xCu_y(110) surfaces. Insets: Configurations of one Zn atom deposition on the Zn(002), Cu(111) and Zn_xCu_y(110) surfaces

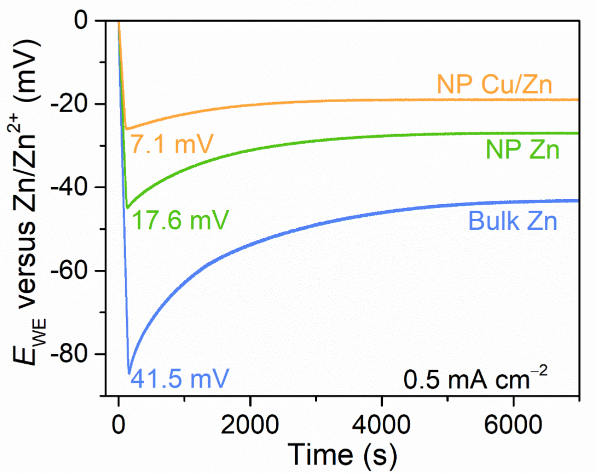

Fig. S12 Comparisons of voltage profiles for galvanostatic Zn deposition on nanoporous Cu/Zn and Zn electrodes, as well as bulk Zn at 0.5 mA cm^{-2}

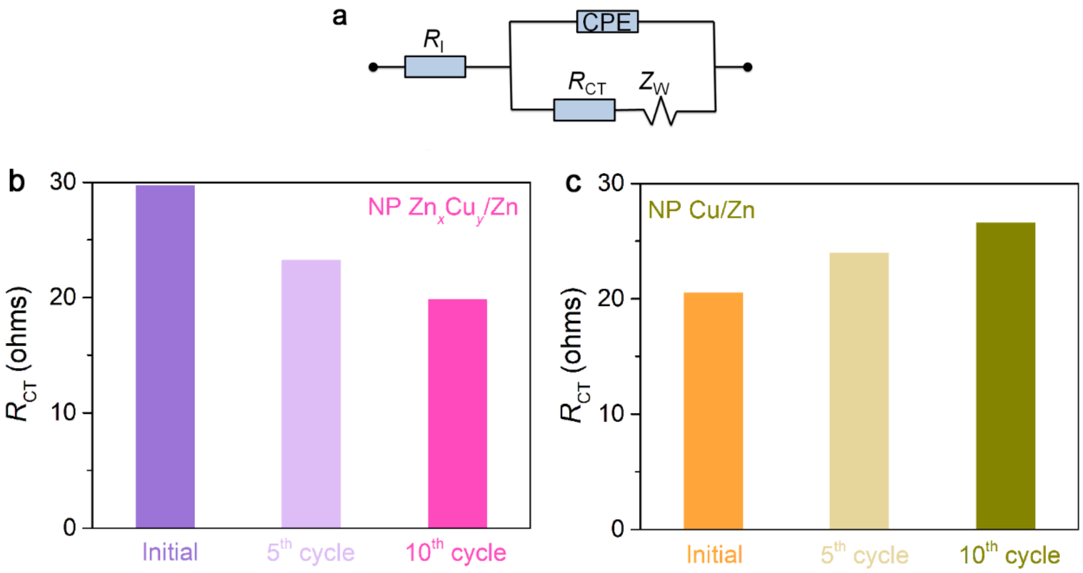

Fig. S13 (**a**) The equivalent circuit with the general descriptors: the intrinsic resistance of both electrolyte and electrode (R_I), the charge transfer resistance (R_{CT}), the constant phase element (CPE) and the slope of the inclined line at flow frequencies corresponding to the Warburg resistance (Z_w). (**b**) Comparison of R_{CT} values for nanoporous Zn_xCu_y/Zn during the Zn stripping/plating for 0, 5 and 10 cycles in 1 M $Zn(OTF)_2$ aqueous electrolyte with SDS additive.

(**c**) Comparison of R_{CT} values for nanoporous Cu/Zn during the Zn stripping/plating for 0, 5 and 10 cycles in 1 M $Zn(OTF)_2$ aqueous electrolyte without SDS additive

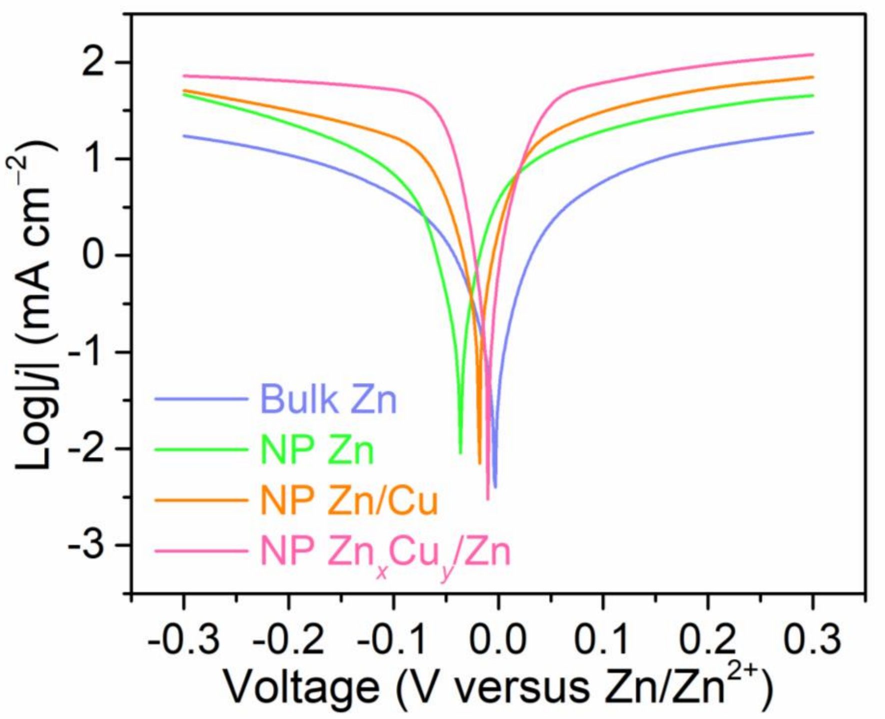

Fig. S14 Tafel polarization curves of nanoporous Zn_xCu_y/Zn, nanoporous Cu/Zn, nanoporous Zn and bulk Zn electrodes

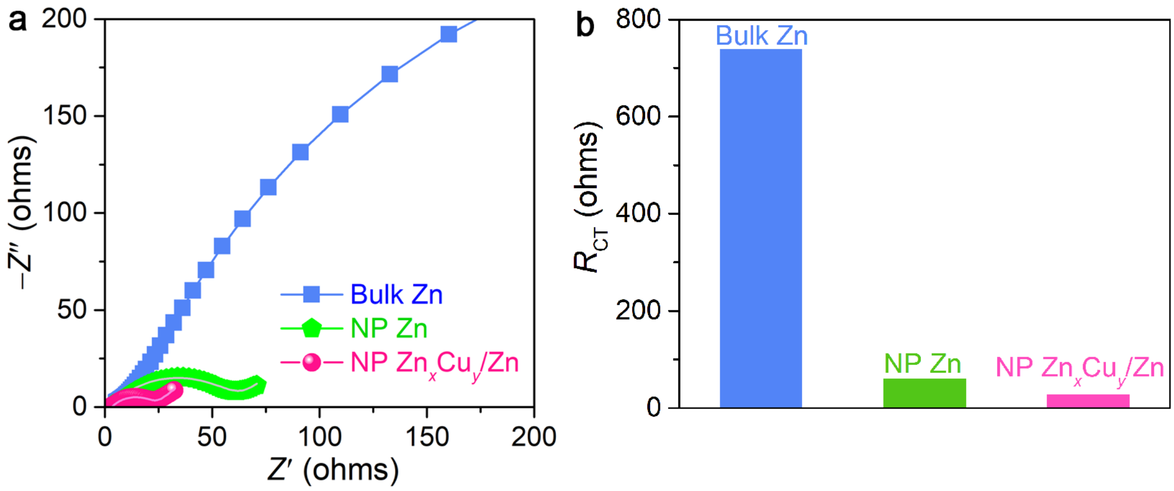

Fig. S15 (**a**) EIS spectra of symmetric cells based on nanoporous Zn_xCu_y/Zn, nanoporous Zn and bulk Zn electrodes. (**b**) Comparison of R_{CT} values for nanoporous Zn_xCu_y/Zn, nanoporous Zn and bulk Zn electrodes

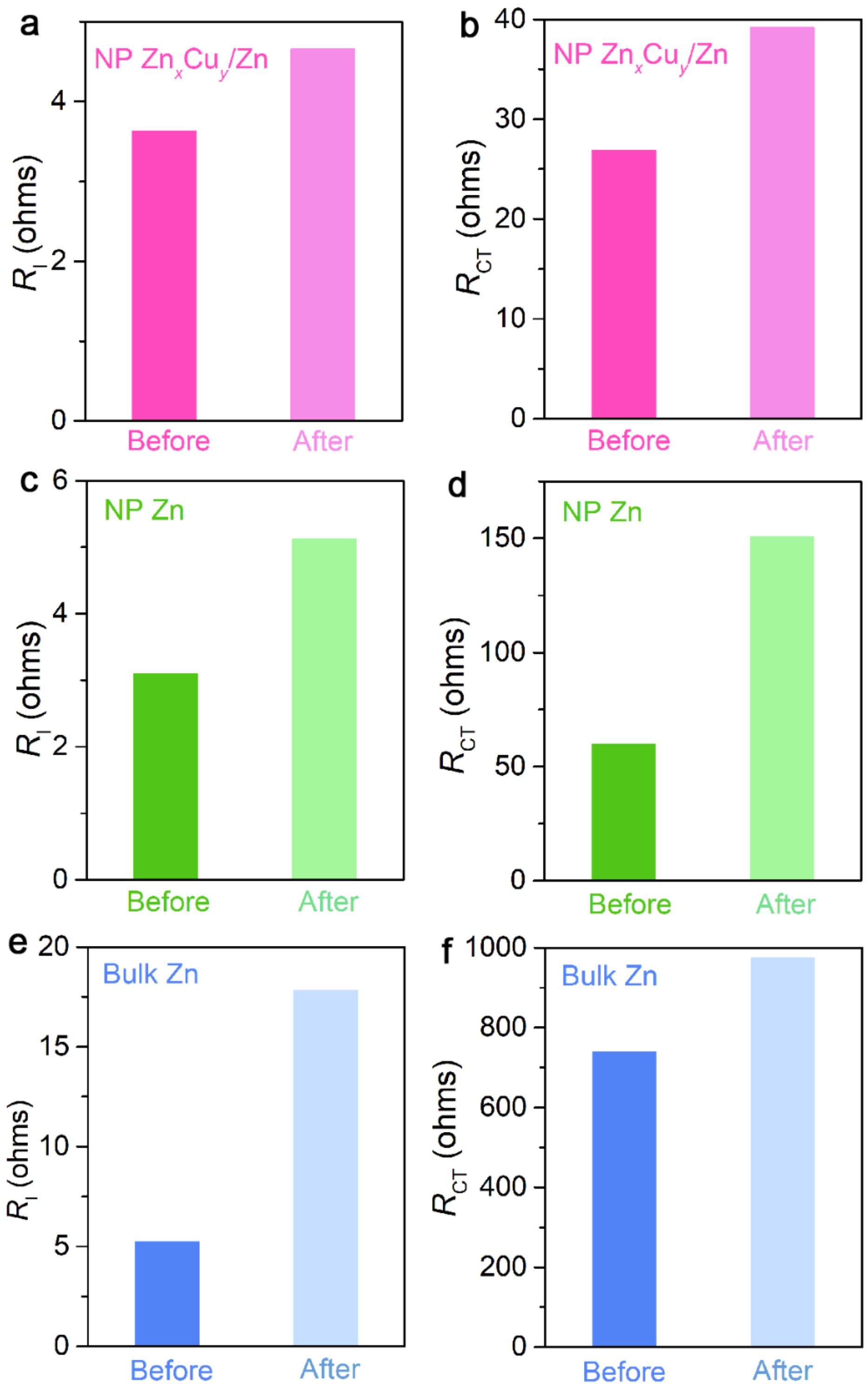

Fig. S16 (**a**, **b**) Comparison of R_I (**a**) and R_{CT} (**b**) values for nanoporous Zn_xCu_y/Zn electrodes before and after 100 cycles of Zn stripping/plating in their symmetric cells. (**c**, **d**) Comparison of R_I (**c**) and R_{CT} (**d**) values for nanoporous Zn electrodes before and after 100 cycles of Zn stripping/plating in their symmetric cell. (**e**, **f**) Comparison of R_I (**e**) and R_{CT} (**f**) values for bulk Zn electrodes before and after 100 cycles of Zn stripping/plating in their symmetric cells

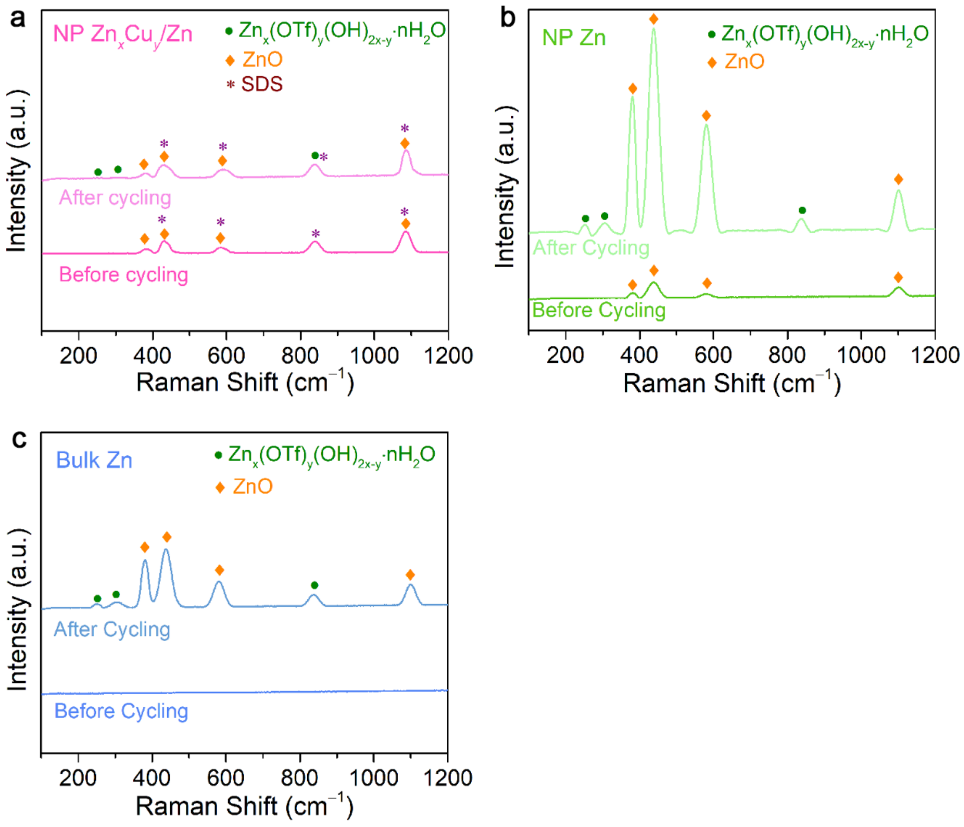

Fig. S17 (**a-c**) Raman spectra of nanoporous Zn_xCu_y/Zn (**a**), nanoporous Zn (**b**) and bulk Zn (**c**) before and after Zn stripping/plating for 100 cycles, during which there generate additional by-products of $Zn_x(OTF)_y(OH)_{2x-y}$ and ZnO

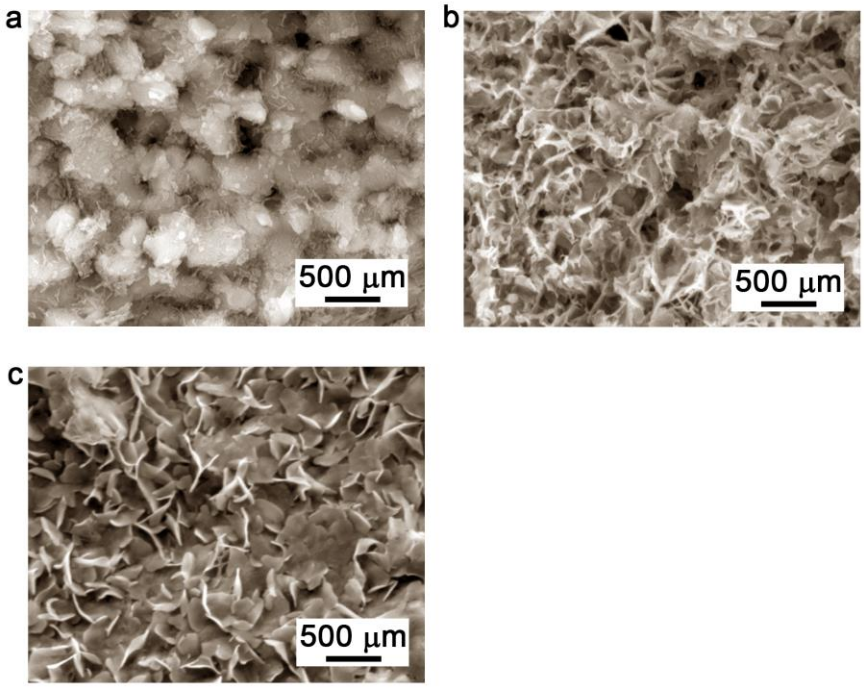

Fig. S18 (**a**) SEM image of nanoporous Zn_xCu_y/Zn electrode after Zn stripping/plating for 500 cycles (1000 hours). (**b**) SEM image of nanoporous Zn electrode after Zn stripping/plating for 150 cycles (300 hours). (**c**) SEM image of bulk Zn foil after Zn stripping/plating for 50 cycles (100 hours)

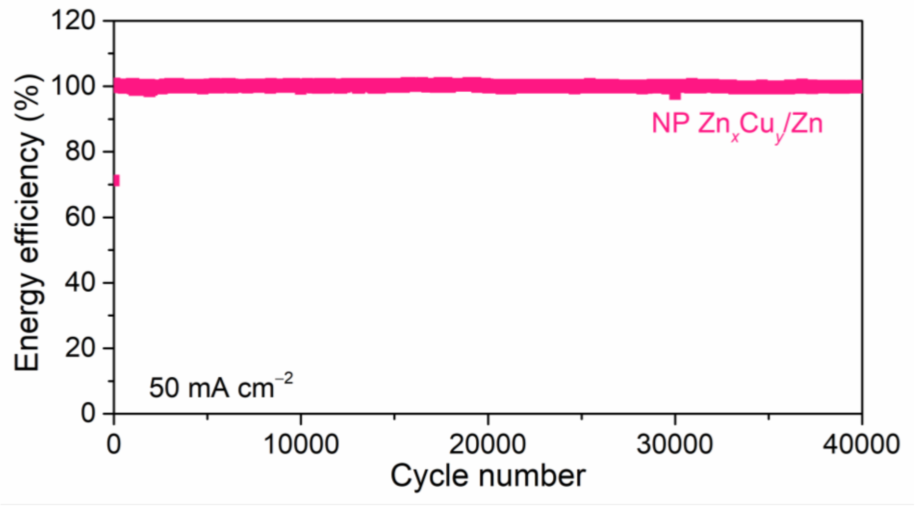

Fig. S19 Energy efficiency of nanoporous Zn_xCu_y/Zn electrodes during long-term cycling of Zn stripping/plating in 1 M $Zn(OTF)_2$ with 1 mM SDS at 50 mA cm^{-2}

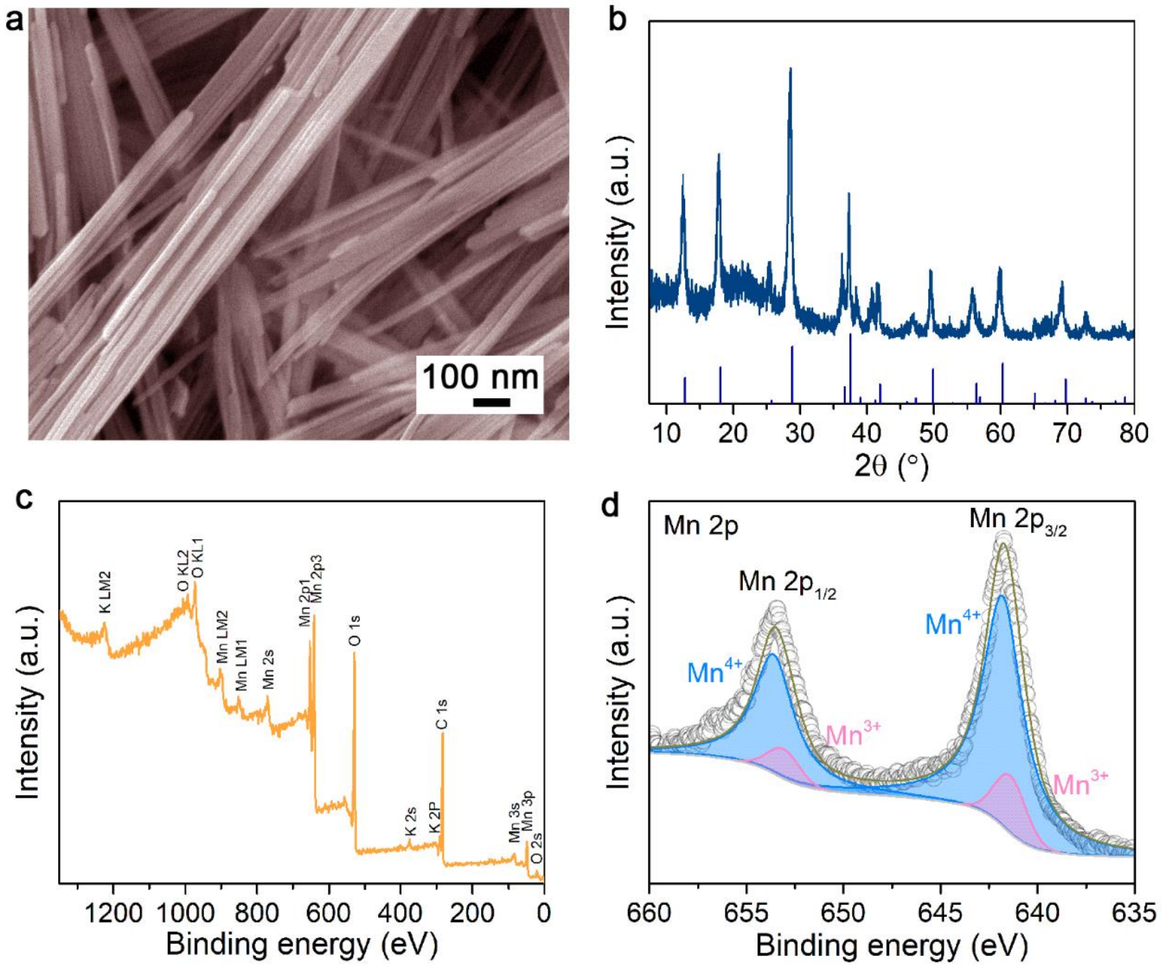

Fig. S20 Structural and chemical characterizations of K_zMnO_2 nanobelts. (**a**) Typical SEM image of as-prepared K_zMnO_2 nanobelts. (**b**) XRD patterns of K_zMnO_2 nanobelts. The line patterns show reference card 44-0141 for tetragonal phase of α-MnO_2 according to JCPDS. (**c**) XPS survey spectrum of as-prepared K_zMnO_2 nanobelts, demonstrating the presence of preintercalated K^+. (**d**) High-resolution Mn 2p XPS spectrum of K_zMnO_2 nanobelts

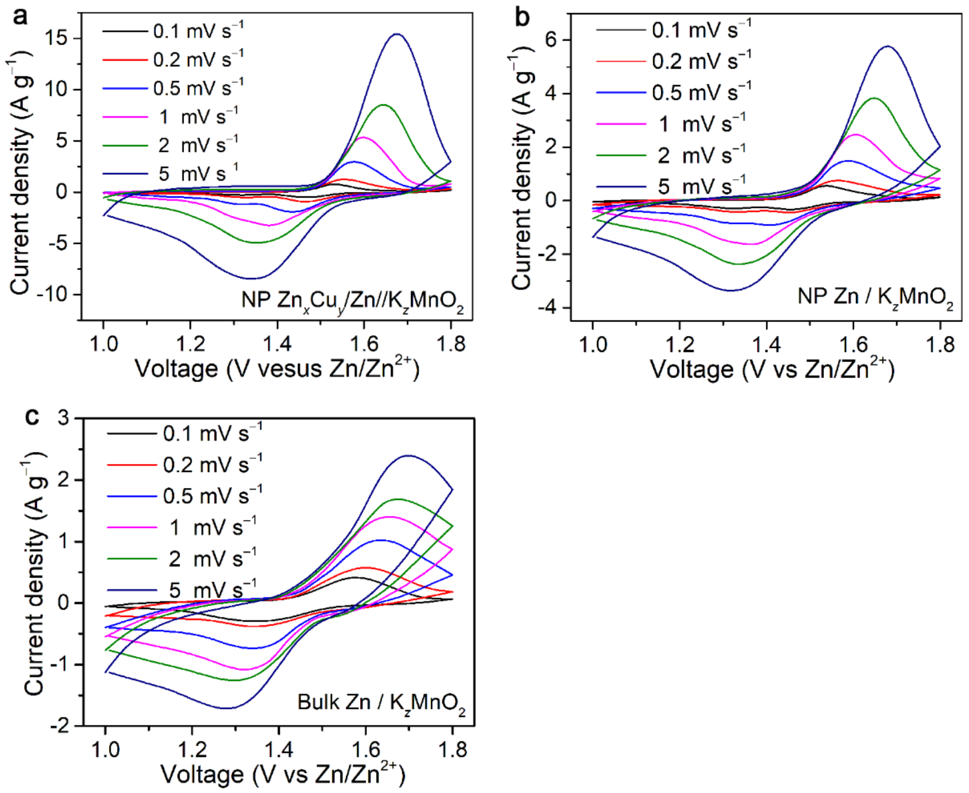

Fig. S21 (**a-c**) CV curves of full Zn-ion batteries of nanoporous $Zn_xCu_y/Zn//K_zMnO_2$ (**a**), nanoporous $Zn//K_zMnO_2$ (**b**) and bulk $Zn//K_zMnO_2$ (**c**) at various scan rates ranging from 0.1 to 5 mV s^{-1}

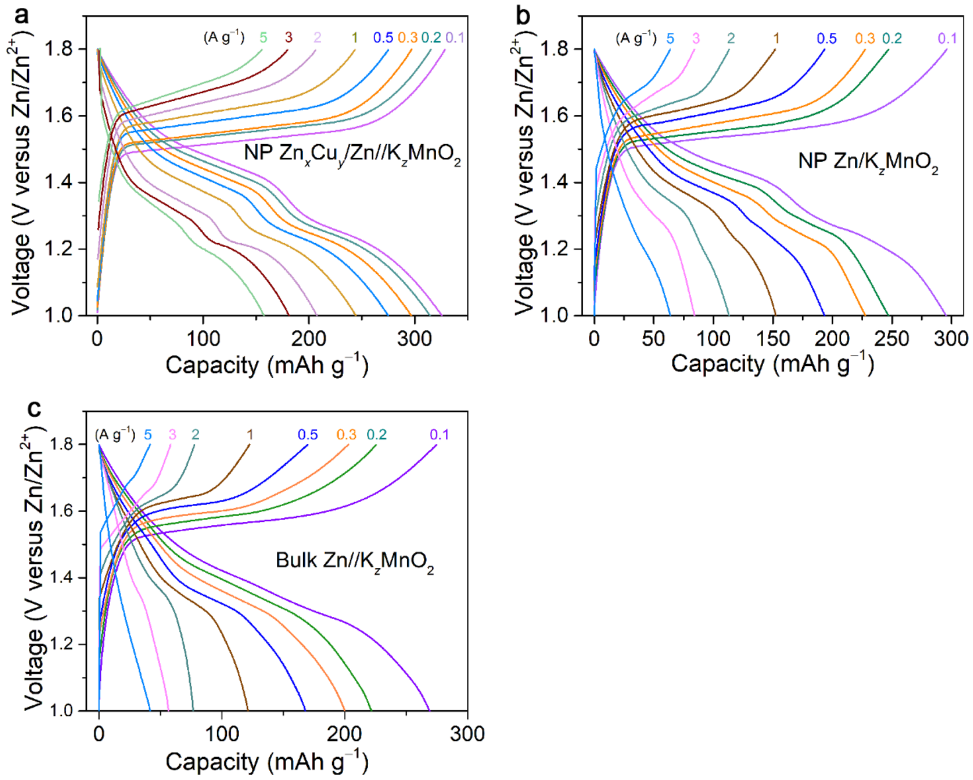

Fig. S22 (**a-c**) Voltage profiles of charge/discharge for full Zn-ion cells of nanoporous $Zn_xCu_y/Zn//K_zMnO_2$ (**a**), nanoporous $Zn//K_zMnO_2$ (**b**) and bulk $Zn//K_zMnO_2$ (**c**) at the specific currents ranging from 0.1 to 5 A g^{-1}. The applied specific current and the achieved specific capacity are calculated according to the loading mass of electroactive K_zMnO_2 in the cathode

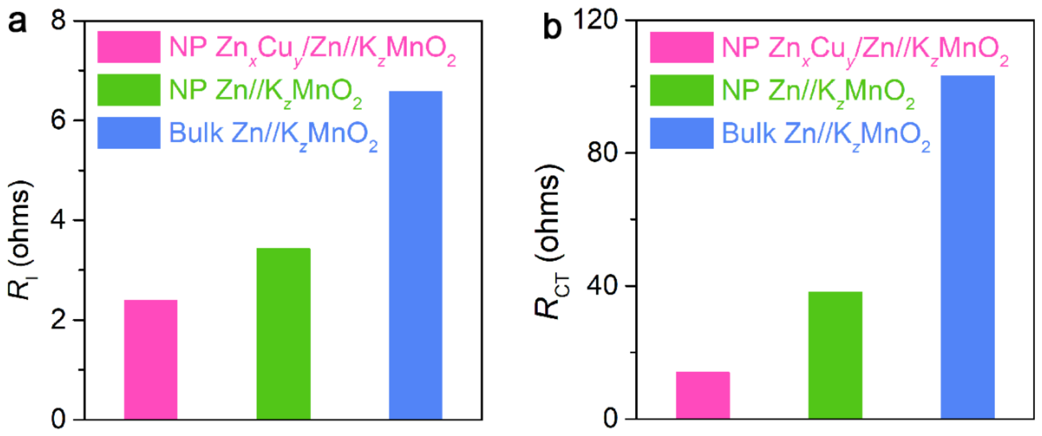

Fig. S23 (**a**,**b**) Comparison of R_I (**a**) and R_{CT} (**b**) values for nanoporous $Zn_xCu_y/Zn//K_zMnO_2$, nanoporous $Zn//K_zMnO_2$ and bulk $Zn//K_zMnO_2$ AR-ZMB devices

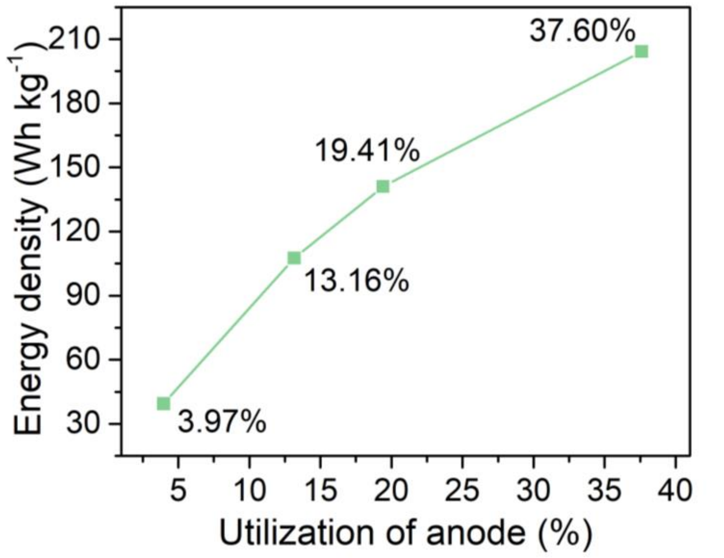

Fig. S24 Dependence of cell-level energy density on Zn utilization of anode in nanoporous $Zn_xCu_y/Zn//K_zMnO_2$ full cells

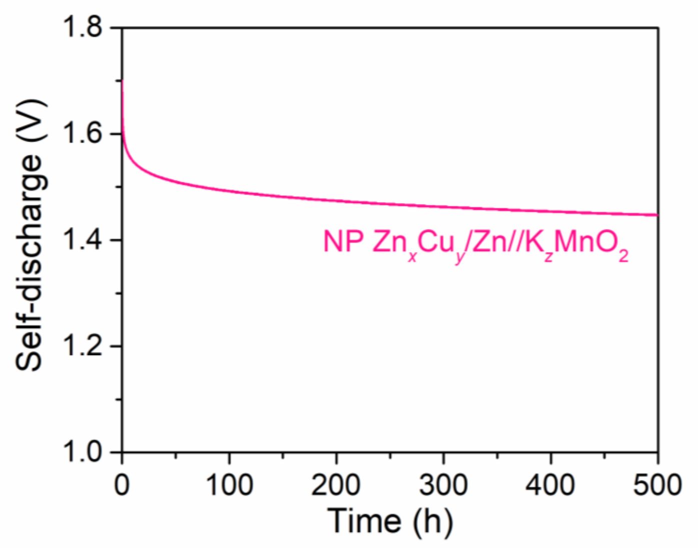

Fig. S25 Self-discharge profile for nanoporous $Zn_xCu_y/Zn//K_zMnO_2$ full cell

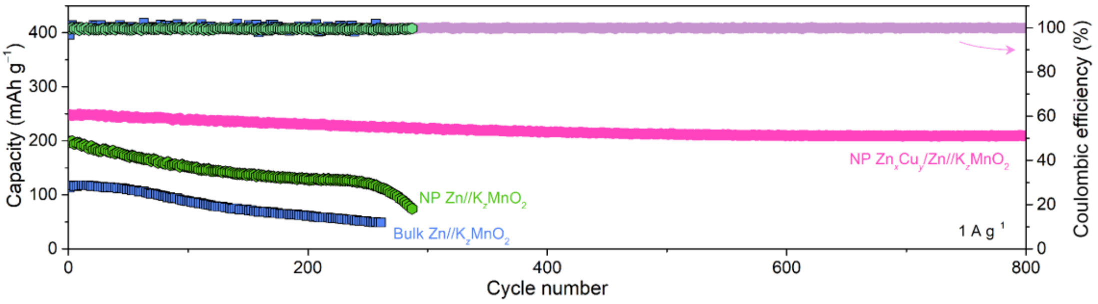

Fig. S26 Comparisons of capacity retentions and coulombic efficiencies for nanoporous Zn_xCu_y/Zn//K_zMnO_2, nanoporous Zn//K_zMnO_2 and bulk Zn//K_zMnO_2 cells in a long-term charge/discharge cycling measurements at the specific current of 1 A g^{-1}
